# Comprehensive analysis of *GASA* family members in the *Malus domestica* genome: identification, characterization, and their expressions in response to apple flower induction

**DOI:** 10.1186/s12864-017-4213-5

**Published:** 2017-10-27

**Authors:** Sheng Fan, Dong Zhang, Lizhi Zhang, Cai Gao, Mingzhi Xin, Muhammad Mobeen Tahir, Youmei Li, Juanjuan Ma, Mingyu Han

**Affiliations:** College of Horticulture, Northwest A&F University, Yangling, Shaanxi 712100 People’s Republic of China

**Keywords:** *GASA* gene, Characterization, Apple, Expression profile, Flower induction

## Abstract

**Background:**

The plant-specific *gibberellic acid stimulated Arabidopsis* (*GASA*) gene family is critical for plant development. However, little is known about these genes, particularly in fruit tree species.

**Results:**

We identified 15 putative *Arabidopsis thaliana GASA* (*AtGASA*) and 26 apple *GASA* (*MdGASA*) genes. The identified genes were then characterized (e.g., chromosomal location, structure, and evolutionary relationships). All of the identified *A. thaliana* and apple GASA proteins included a conserved GASA domain and exhibited similar characteristics. Specifically, the *MdGASA* expression levels in various tissues and organs were analyzed based on an online gene expression profile and by qRT-PCR. These genes were more highly expressed in the leaves, buds, and fruits compared with the seeds, roots, and seedlings. *MdGASA* genes were also responsive to gibberellic acid (GA_3_) and abscisic acid treatments. Additionally, transcriptome sequencing results revealed seven potential flowering-related *MdGASA* genes. We analyzed the expression levels of these genes in response to flowering-related treatments (GA_3_, 6-benzylaminopurine, and sugar) and in apple varieties that differed in terms of flowering (‘Nagafu No. 2’ and ‘Yanfu No. 6’) during the flower induction period. These candidate *MdGASA* genes exhibited diverse expression patterns. The expression levels of six *MdGASA* genes were inhibited by GA_3_, while the expression of one gene was up-regulated. Additionally, there were expression-level differences induced by the 6-benzylaminopurine and sugar treatments during the flower induction stage, as well as in the different flowering varieties.

**Conclusion:**

This study represents the first comprehensive investigation of the *A. thaliana* and apple *GASA* gene families. Our data may provide useful clues for future studies and may support the hypotheses regarding the role of GASA proteins during the flower induction stage in fruit tree species.

**Electronic supplementary material:**

The online version of this article (10.1186/s12864-017-4213-5) contains supplementary material, which is available to authorized users.

## Background

There has recently been an increase in the number of studies regarding low-molecular weight peptides. Gibberellic acid stimulated Arabidopsis (GASA), which is a cysteine-rich peptide, is an example of a low-molecular weight peptide important for plant growth and development.

The *GAST1* gene, which was first identified in the *gib1* tomato mutant, belongs to the first identified *GASA* gene family [1]. Many *GASA* homologs have recently been identified in *Arabidopsis thaliana*, tomato, rice, potato, wheat, and *Petunia hybrida* [2–6]*.* The GASA proteins typically consist of 80–270 amino acids, but there are exceptions (e.g., AtGASA14)*.* The *GASA* genes encode small proteins with the following three domains [7]: (1) an N-terminal signal peptide with 18–29 amino acids, (2) a highly variable hydrophilic region with 7–31 polar amino acid residues, and (3) a C-terminal GASA domain consisting of 60 amino acids, typically including 12 cysteine residues [8, 9]. Additionally, previous studies revealed that peptides with a mutated or missing GASA domain are non-functional [10, 11].

Clarifying the subcellular localization of proteins may provide important clues regarding function. Most identified GASA proteins are reportedly localized in the cell wall or apoplast, with the signal peptide serving a critical function related to protein trafficking and localization [3, 4, 12]. For example, AtGASA4 and AtGASA6 are normally present at the cell periphery, but localize in the nucleus if lacking the signal peptide [9]. Additionally, two other GASA proteins, GIP2 and GIP5, accumulate in the cell well in petunia [3]. Meanwhile, an earlier study involving recombinant OsGASR-GFP proteins revealed that OsGASR1 and OsGASR2 localize to the apoplast or cell wall [4]. What’s more, OsGSR1 was detected in the plasma membrane, cytoplasm, and nucleus [13].

In plants, some GASA proteins have been functionally characterized and reportedly affect several processes during growth and development, including defense responses against pathogens and fungi, and stress resistance [10, 14–17]. Other studies have confirmed that GASA proteins influence hormone-related processes such as seed germination, floral development, stem elongation, root development, and signal transduction [3, 10, 12, 13, 18, 19]. For example, OsGSR1 can interact with DIM/DWF1, which is a brassinosteroid synthetase, to influence brassinosteroid signaling in rice [13]. Additionally, Most GASA family members are involved in gibberellic acid (GA_3_) signaling [4, 18], while some are also associated with abscisic acid (ABA), naphthaleneacetic acid, and indole-3-acetic acid signaling. For example, AtGASA2/3, AtGASA5, and AtGASA14 have been linked to ABA signaling [9]. GASA family members may exhibit opposing functions. Researchers have confirmed that AtGASA4 promotes flowering, while AtGASA5 induces the opposite effect [7, 12, 20]. Additionally, in *Gerbera hybrida*, the proteins encoded by *GEG* and *PRGL*, which are two *GASA* homologs, have different functions regarding floral development. Earlier studies demonstrated that GEG inhibits petal elongation, while PRGL induces petal elongation [21, 22]. Among their biological activities, their effect on flowering is one of the most prominent. Plants over-expressing *AtGASA5* reportedly exhibit a late-flowering phenotype as well as down-regulated expression of *FT* and *LFY*, but up-regulated *FLC* expression [12]. In contrast, *AtGASA4* expression promotes floral development [20]. Additionally, overexpressing *FaGAST2* showed delayed growth in strawberry [23]. However, very little is known about GASA genes in woody plants as well as apple.

In contrast to the *GASA* genes in the model plant, *A. thaliana*, as well as in other plant species, which have been studied, little is known about the *GASA* genes in perennial woody species. Apple, as an important fruit tree species, is widely cultivated in temperate regions. The induction of flowering is an important consideration for apple producers and breeders. Most widely grown apple cultivars have a long juvenile period and exhibit poor flower bud development, which is problematic for the apple industry [24, 25]. Flower induction in apple trees is mediated by a complex biological process involving several important gene families, including *SPL*, *MADs-box*, and *IDD* [24, 26, 27]. Thus, identifying apple *GASA* family members and characterizing their potential roles will undoubtedly be useful. The sequenced apple genome [28] enables a whole-genome search for *GASA* genes as well as candidate genes responsible for inducing flower bud development. In this study, we first identified the *GASA* genes in the *A. thaliana* and apple genomes. We then analyzed the gene structures and classifications as well as phylogenetic relationships to characterize the apple *GASA* genes. Furthermore, an analysis of tissue-specific and flowering-related gene expression revealed candidate *GASA* genes associated with flower induction. To our best understanding, this study represents the first comprehensive analysis of *A. thaliana* and apple *GASA* genes. Our data may serve as a valuable resource for future studies of *GASA* genes related to flowering in apple as well as in other fruit tree species.

## Results

### Genome-wide identification of *Arabidopsis thaliana* and *Malus domestica GASA* genes

**Fig. 1:**
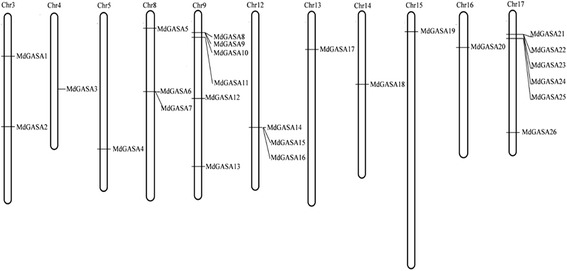
Locations of *MdGASA* genes on each apple chromosome

Thirteen *GASA* genes were previously identified in the *A. thaliana* TAIR7.0 genome [20]. Another two *AtGASA* genes (AT3g10185 and AT1g10588) were then verified and replenished in the *Arabidopsis* genome. These two added new genes were named *AtGASA14* and *AtGASA15*. And these 15 *AtGASA* genes all shared conserved 12 cysteines (Additional file 1). To identify apple *GASA* genes, a BLASTP search of the apple genome was completed with the 15 AtGASA protein sequences used as queries. Furthermore, the 26 putative apple GASA genes were manually checked and confirmed using the conserved domain database (https://www.ncbi.nlm.nih.gov/Structure/cdd/wrpsb.cgi) (Table 1). They were named according to their chromosomal locations (*MdGASA1–26*) (Fig. 1). The 26 *MdGASA* genes were located in 11 chromosomes in the apple genome. The chromosomes 9 and 17 contain most of the genes with 6 genes each, while the chromosomes 4, 5, 13, 14, 15, and 16 contain 1 copy each. Chromosome 8 and 12 contained three genes (Fig. 1).

**Fig. 2:**
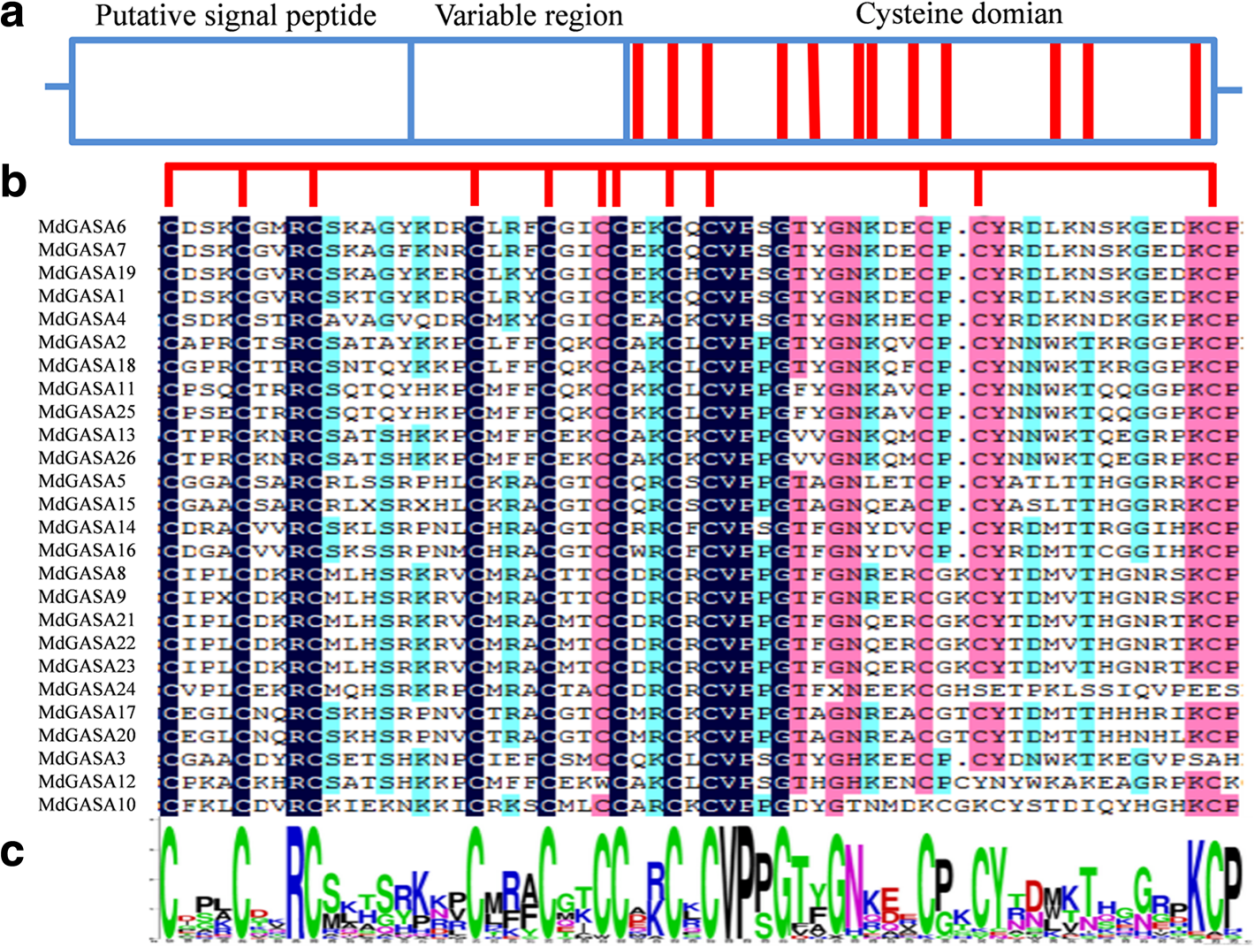
Alignment of the GASA domain from MdGASA proteins. **a** Analysis of GASA protein structures, red column represented their conserved twelve cysteines. **b** Multiple alignments of the MdGASA protein sequences. Their conserved GASA domains were indicated. **c** Sequence logo analysis of the conserved GASA domains. Each stack represented their amino acids

The full MdGASA protein sequences were aligned to examine whether a GASA domain was present (Fig. 2b). All of the putative MdGASA proteins shared a conserved GASA domain, except for MdGASA24 and MdGASA10, whose GASA domains were mutated by the insertion of several amino acids.

### Gene characterization and structure analysis of MdGASA

**Fig. 3:**
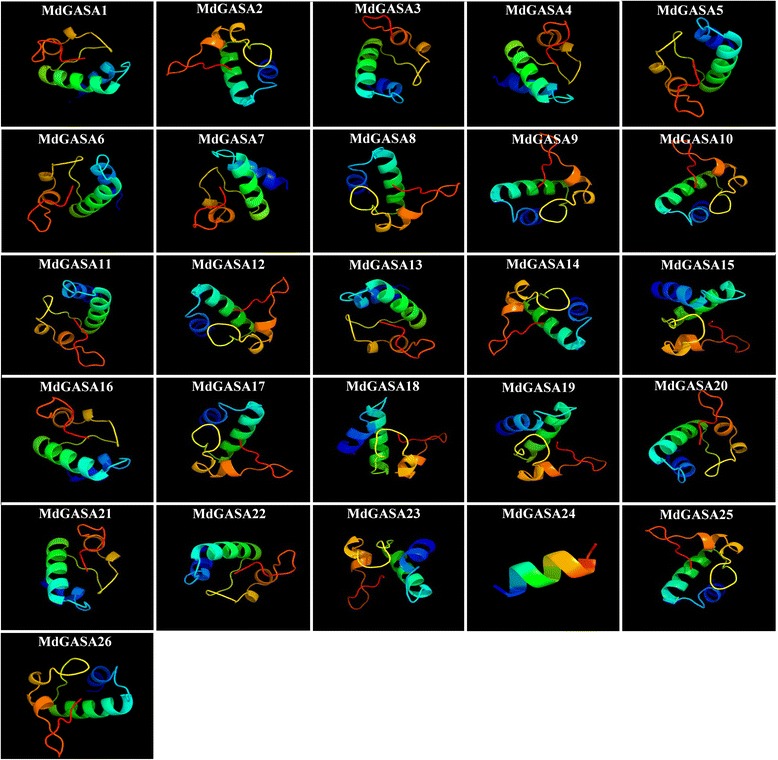
Predicted dimensional structures of MdGASA proteins

Protein characteristics, including molecular weight, isoelectric point, instability index, grand average of hydropathicity (GRAVY), major amino acid content, and aliphatic index, were analyzed with the ExPASy program. The molecular weight of the analyzed GASA proteins ranged from 9.44 (AtGASA8) to 33.97 (MdGASA24). Additionally, the molecular weight of most of the GASA proteins was less than 13 (i.e., low-molecular weight peptides). Moreover, the isoelectric point ranged from 7.41(AtGASA1) to 10.14 (MdGASA24) (Table 2). Most of the GASA proteins were observed to be unstable, with instability index values greater than 40. The exceptions were AtGASA5, AtGASA6, AtGASA9, AtGASA10, AtGASA12, MdGASA1, MdGASA4, and MdGASA7. According to the GRAVY values, the GASA proteins were hydrophilic, except for AtGASA6, AtGASA610, AtGASA11, AtGASA12, MdGASA5, MdGASA6, MdGASA14, MdGASA15, and MdGASA16. Meanwhile, the aliphatic index values ranged from 84.65 (AtGASA12) to 41.67 (MdGASA11). In terms of amino acid content, Cys, Lys, and Leu were the predominant residues, while Ser, Asp, Val, Pro, and Thr were also detected among the *A. thaliana* and apple GASA proteins. We also analyzed the transmembrane helices of all MdGASA proteins. At least one transmembrane segment was detected for MdGASA3, MdGASA6, MdGASA11, MdGASA12, MdGASA13, MdGASA18, MdGASA20, MdGASA25, and MdGASA26 (Additional file 2). The predicted protein structures for all MdGASA proteins (Fig. 3, Additional file 3) revealed the presence of α helices, β sheets, extended strands, and random coils. Of these structures, random coils were the most abundant, while β sheets were the least common. Moreover, the random coils were larger than the α helices.

**Fig. 4:**
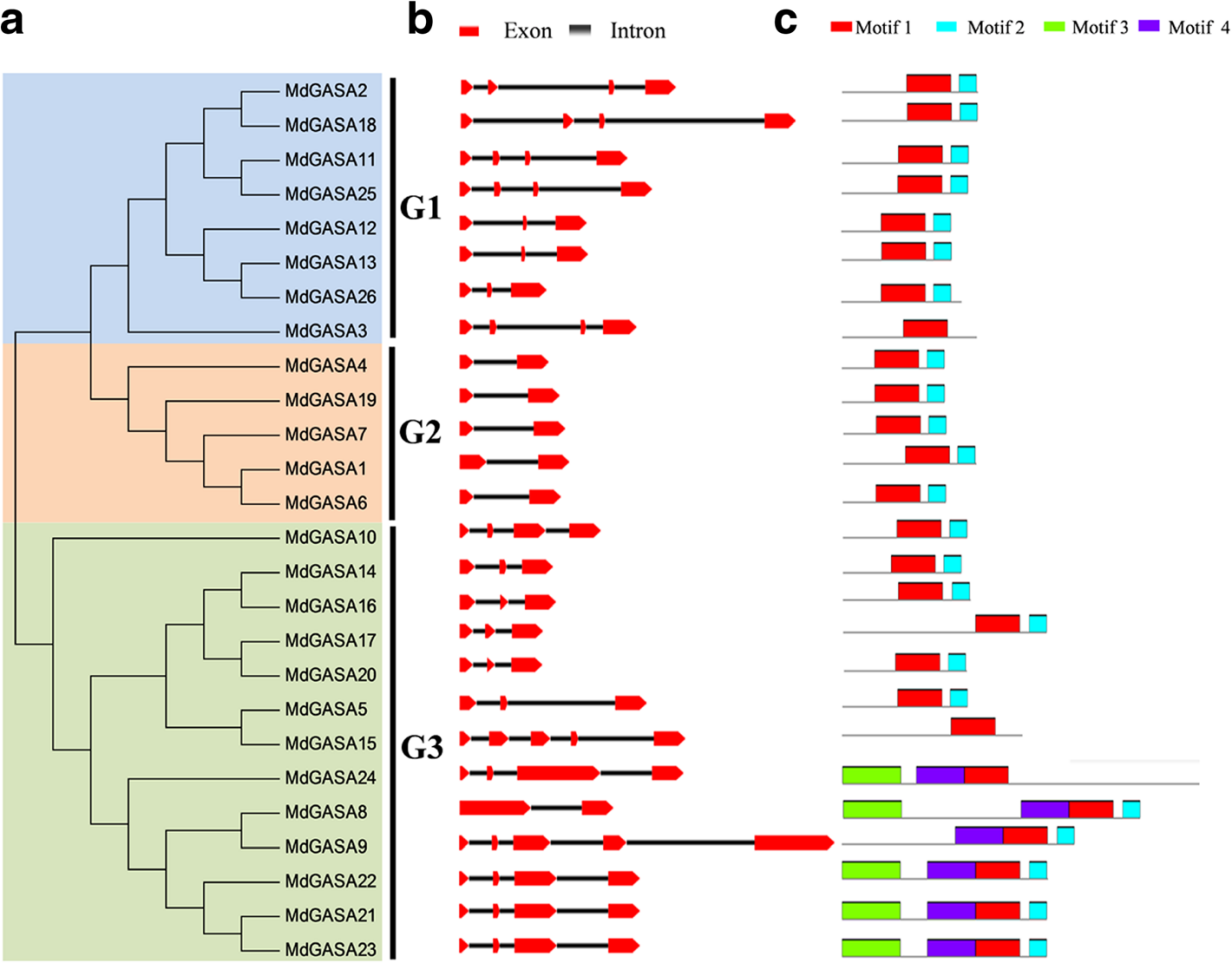
Analysis of *MdGASA* gene structures. **a** An unrooted phylogenetic tree constructed based on MdGASA protein sequences. **b** Exon-intron composition analysis, red boxes and black line were exon and intron positions, respectively. **c** Conserved motifs analysis, details motifs can be seen Fig. S2

Exon-intron structures were generated based on the annotated apple genome using the Gene Structure Display Server program. The *MdGASA* genes within each group shared conserved genetic structures (Fig. 4b). For example, the Group 2 genes, which included *MdGASA4*, *MdGASA19*, *MdGASA7*, *MdGASA1*, and *MdGASA6*, all contained one intron and two exons. Meanwhile, *MdGASA2*, *MdGASA18*, *MdGASA11*, and *MdGASA25* were highly conserved and comprised four exons and three introns. Similar results were observed for the Group 3 genes (*MdGASA14*, *MdGASA16*, *MdGASA17*, and *MdGASA20*), which consisted of three exons and two introns. Furthermore, we detected four conserved protein motifs among the MdGASA proteins (Fig. 4c, Additional file 4). Members of Groups 1 and 2 carried motifs 1 and 2 except MdGASA3. G1 and G2 all shared motif 1 and motif 2. However, in Group 3, MdGASA15 contained only motif 1, while, MdGASA8, MdGASA21, MdGASA22, and MdGASA23 consisted of all four motifs.

### Analysis of synteny and evolutionary relationships among *GASA* genes

**Fig. 5:**
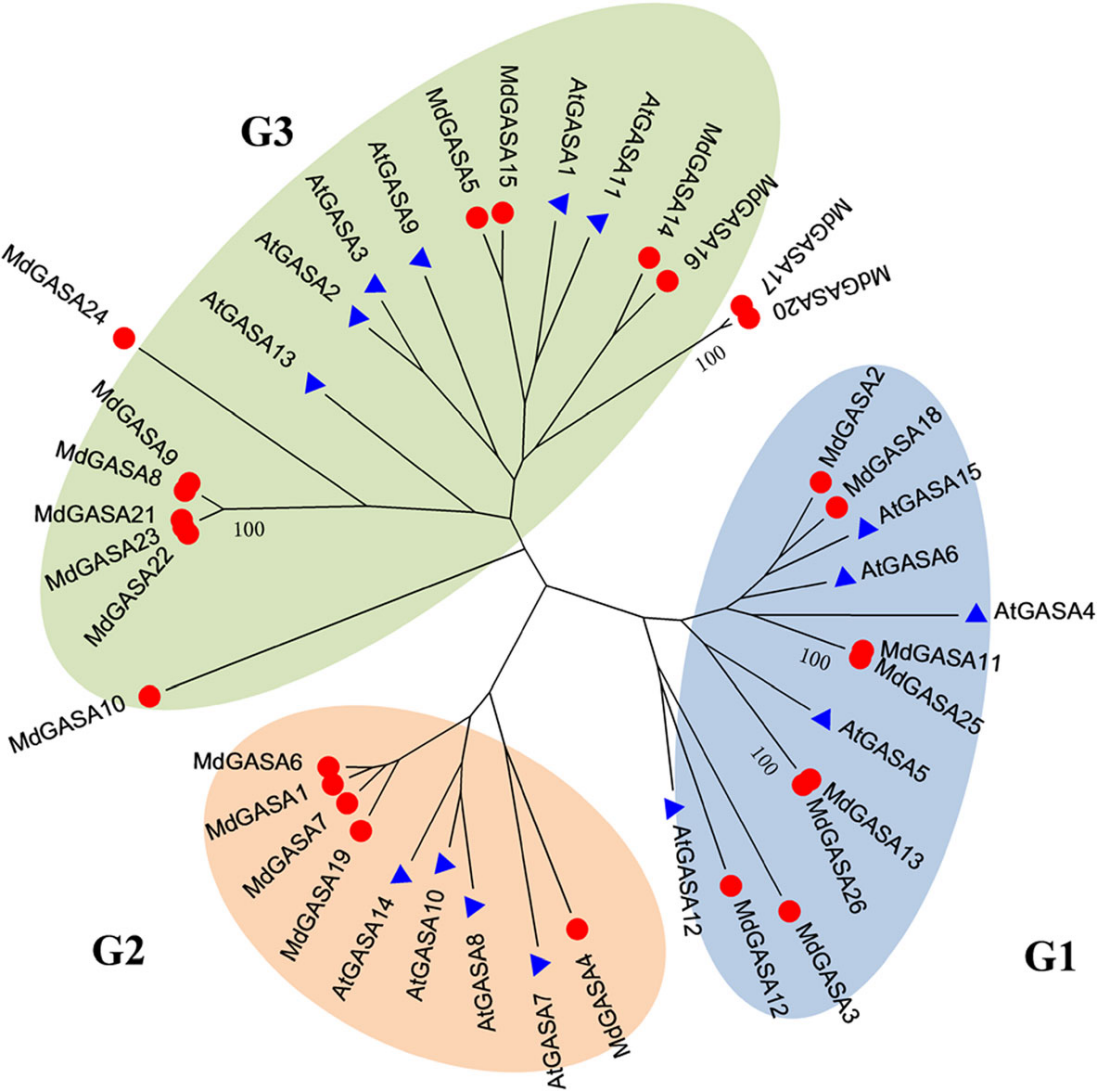
Phylogenetic analysis of apple and *Arabidopsis thaliana GASA* genes. Protein designations: *Arabidopsis* (At, blue triangle) and apple (Md, red circle)

To clarify the evolutionary relationships among *GASA* genes, we constructed a phylogenetic tree based on the *A. thaliana* and apple GASA protein sequences. According to the phylogenetic tree (Fig. 5), the *A. thaliana* and apple *GASA* genes were classified into three groups, with Groups 1, 2, and 3 consisting of 13, 9, and 19 GASA genes, respectively. Eight apple genes (*MdGASA2*, *MdGASA18*, *MdGASA11*, *MdGASA25*, *MdGASA13*, *MdGASA26*, *MdGASA3*, and *MdGASA12*) were clustered in Group 1, while five genes (*MdGASA4*, *MdGASA9*, *MdGASA7*, *MdGASA1*, and *MdGASA6*) were clustered in Group 2 and 13 genes (*MdGASA10*, *MdGASA21*, *MdGASA22*, *MdGASA23*, *MdGASA8*, *MdGASA9*, *MdGASA24*, *MdGASA5*, *MdGASA15*, *MdGASA14*, *MdGASA16*, *MdGASA17*, and *MdGASA20*) were clustered in Group 3.

**Fig. 6:**
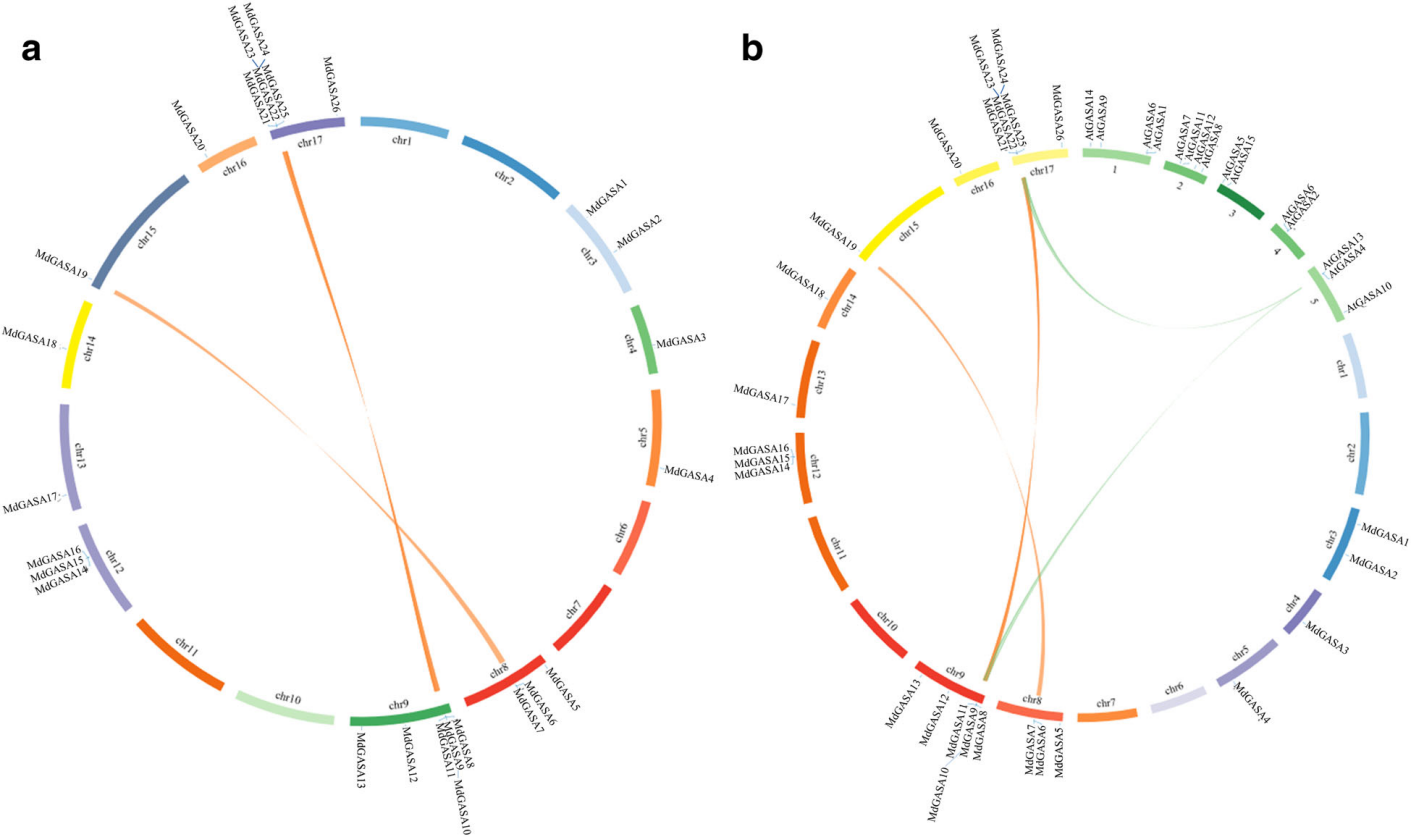
Analysis of evolutionary relationships among *GASA* gene family members. Relative positive positions were depicted according to the apple chromosomes, colored lines were syntenic regions of the apple genome. (**b**) Synteny analysis of *GASA* genes between Arabidopsis and apple, relative positive positions were depicted according to the apple and *Arabidopsis* chromosomes, colored lines were syntenic regions of apple and *Arabidopsis* genome

To characterize the expansion patterns of the *MdGASA* genes, a diagram prepared with the Circos program was used to examine the duplicated blocks in the apple genome. Finally, two pairs of *MdGASA* genes (*MdGASA7–MdGASA19* and *MdGASA9–MdGASA22*) were distributed among four chromosomes (Fig. 6a). Additionally, these duplicated *MdGASA* genes were from chromosomes with many genes, including chromosomes 8, 9, and 17. The exception was *MdGASA19*, which was located on chromosome 15.

Additional diagrams were prepared using the Circos program to evaluate the evolutionary relationships among *A. thaliana* and apple *GASA* genes (Fig. 6b). Two *A. thaliana*–apple *GASA* gene pairs (*AtGASA13*–*MdGASA9* and *AtGASA4*–*MdGASA22*) were identified from three chromosomes. These two paired genes were detected in syntenic genomic regions. Additionally, both of the duplicated *AtGASA* genes were located on chromosome 5, while the two duplicated *MdGASA* genes were located on chromosomes 9 and 17.

### *MdGASA* expression patterns in different tissues or organs

**Fig. 7:**
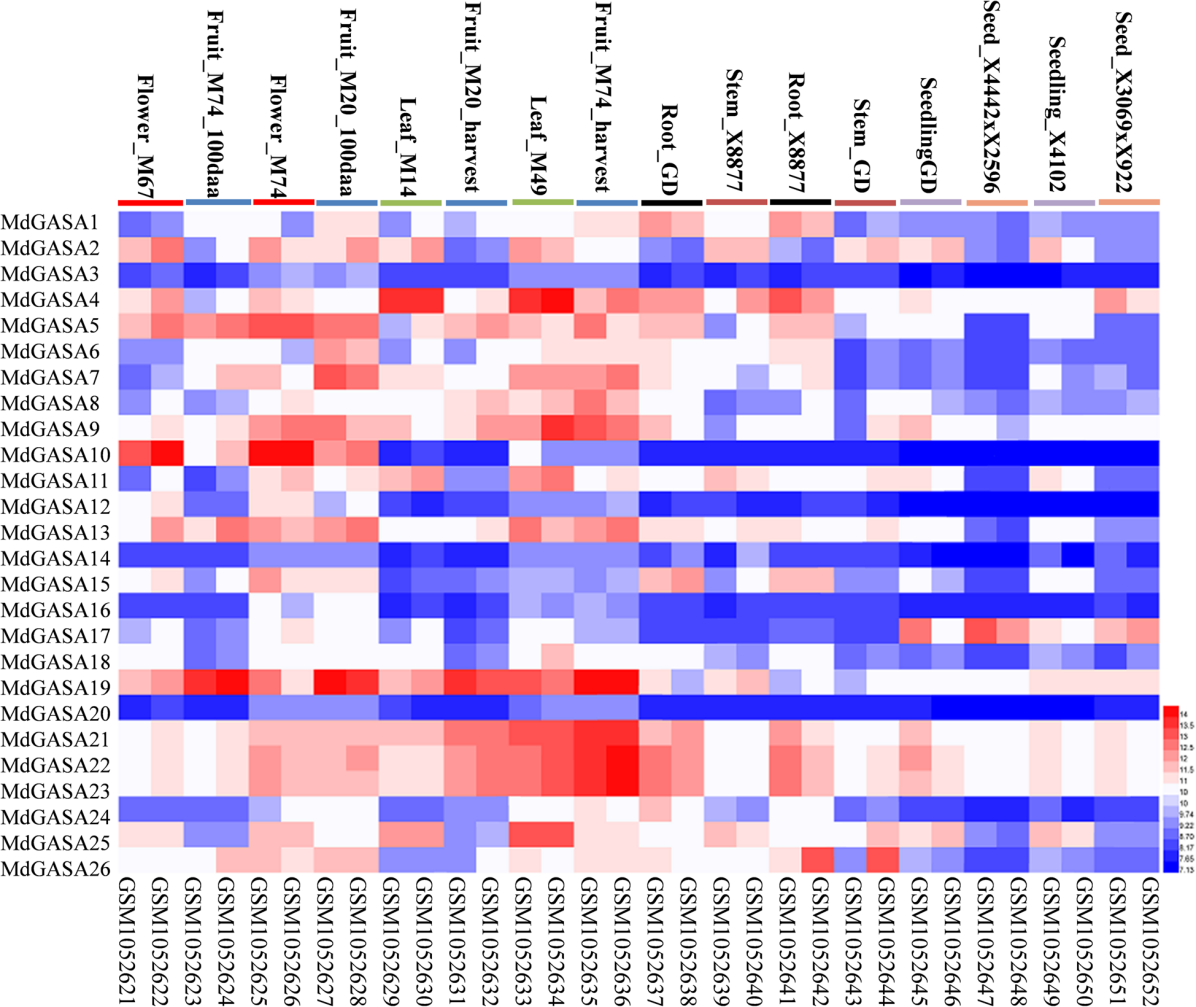
*MdGASA* gene expression profiles in different tissues and in different apple varieties. Relative expression profiles are based the online database (E-GEOD-42873)

While *A. thaliana GASA* genes have been relatively well characterized, little is known about the expression of apple *GASA* genes. Thus, we systematically investigated their expression patterns in different tissues or organs using an online ArrayExpress database (E-GEOD- GSE42873) and a quantitative real-time polymerase chain reaction (qRT-PCR). Seven tissues or organs (leaves, flowers, fruits, seeds, stems, roots, seedlings) from 10 apple varieties (‘M67’, ‘M74’, ‘M20’, ‘M14’, ‘M9’, ‘M74’, ‘GD’, ‘X8877’, and two hybrids) were analyzed. The 26 candidate *MdGASA* genes exhibited diverse expression patterns among the various tissues (Fig. 7). All of the *MdGASA* genes were more highly expressed in the flowers, fruits, and leaves than in the stems and seedlings (Fig. 7). Moreover, *MdGASA3*, *MdGASA14*, and *MdGASA20* were hardly expressed in all tissues and varieties, while *MdGASA16* was highly expressed only in ‘M74’ flowers and ‘M20’ fruits.

**Fig. 8:**
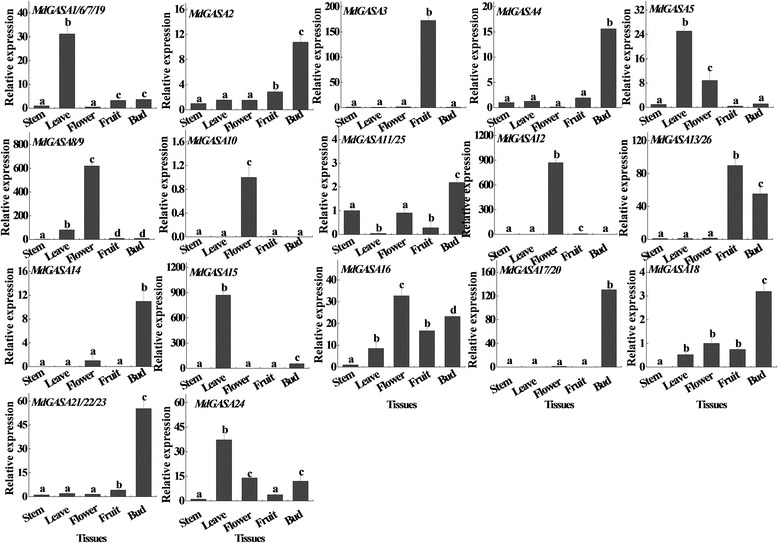
Analysis of *MdGASA* expression levels in different ‘Nagafu No. 2’ tissues. Each value represents the mean ± standard error of three biological replicates. Means followed by small letters are significantly different at the 0.05 level

To further analyze the potential MdGASA functions related to apple growth and development, different tissues (stems, leaves, flowers, fruits, and buds) were collected from ‘Nagafu No. 2’ trees. Because the *MdGASA* genes were extremely short and included a highly conserved GASA domain, 17 primer pairs were only designed to analyze expression levels [24, 26]. We observed diverse *MdGASA* expression patterns in ‘Nagafu No. 2’ tissues (Fig. 8). For example, *MdGASA3* and *MdGASA13/26* were highly expressed in fruits, while *MdGASA1*/*6*/*7*/*19*, *MdGASA5*, *MdGASA15*, and *MdGASA24* expression levels were high in leaves. In contrast, *MdGASA2*, *MdGASA4*, *MdGASA11/25*, *MdGASA14*, *MdGASA17/20*, *MdGASA18*, and *MdGASA21/22/23* were highly expressed in buds.

### Effect of GA_3_ and ABA treatments on the expression of MdGASA genes in apple leaves

To elucidate the effects of phytohormones on *MdGASA* expression, trees were treated with GA_3_ and ABA, and leaves were collected at 3, 6, 12, and 24 h after treatments. As shown in Additional file 5, the *MdGASA* genes were responsive to exogenously applied GA_3_ and ABA, with the exception of *MdGASA4*, *MdGASA17/20*, and *MdGASA24*. *MdGASA11/25*, *MdGASA15*, and *MdGASA21/22/23*, were down-regulated by GA_3_ during the sampling period or at most time points. Meanwhile, *MdGASA13/26* expression was inhibited at all time points. Furthermore, the largest increase in GA_3_-induced *MdGASA* expression was observed for *MdGASA18* at 3 h after treatment.

### *MdGASA* expression patterns in response to GA_3_ and 6-benzylaminopurine treatments during the flower induction period

**Fig. 9:**
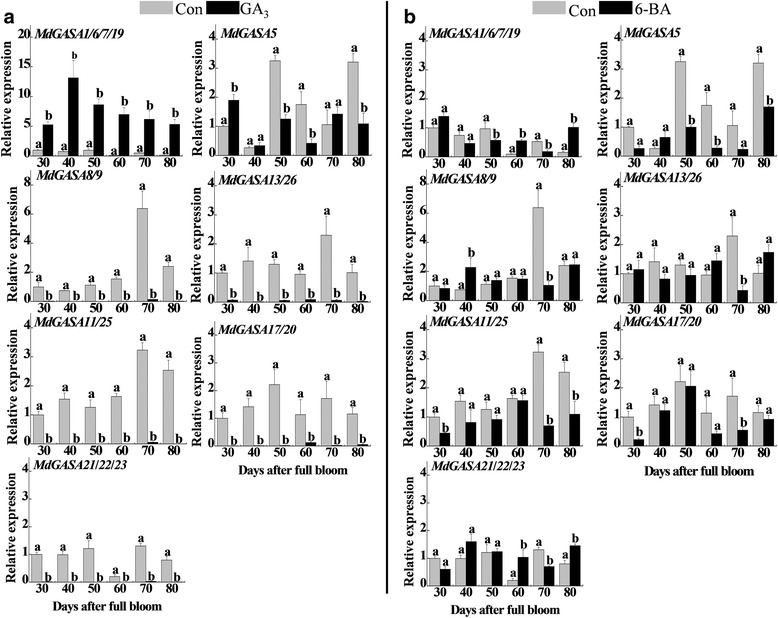
Analysis of flowering-related *MdGASA* expression levels in response to GA_3_ (**a**) and 6-BA (**b**) treatments. Samples were collected at 30, 40, 50, 60, 70, and 80 days after full bloom (DAFB), with water used as a control. Each value represents the mean ± standard error of three replicates. Means followed by small letters are significantly different at the 0.05 level

To assess the potential effects of *MdGASA* expression induced by GA_3_ or 6-benzylaminopurine (6-BA) on apple flower induction, the transcript levels of seven candidate genes were investigated (Additional file 6) [29]. As shown in Fig. 9a, *MdGASA1*/*6*/*7*/*19* expression was up-regulated by GA_3_ at all time points. Additionally, *MdGASA5* expression was initially up-regulated, but was subsequently down-regulated during the flower induction period. The transcript levels for the other candidate genes were down-regulated by exogenously applied GA_3_. In contrast, *MdGASA* expression patterns varied over time in response to 6-BA (Fig. 9b). For example, *MdGASA1*/*6*/*7*/*19* expression was inhibited only at 40, 50, and 70 days after full bloom (DAFB). In contrast, *MdGASA5* expression was up-regulated by 6-BA except at 40 DAFB. Moreover, *MdGASA8/9* expression was inhibited only at 30 and 70 DAFB, while *MdGASA13*/*26* expression was inhibited at 40, 50, and 70 DAFB. Furthermore, *MdGASA21*/*22*/*23* expression was up-regulated except at 30 and 70 DAFB, while *MdGASA11*/*25* and *MdGASA17*/*20* expression was inhibited by 6-BA at all time points.

### *MdGASA* expression patterns in response to sugar treatments and in different flowering varieties during the flower induction period

**Fig. 10:**
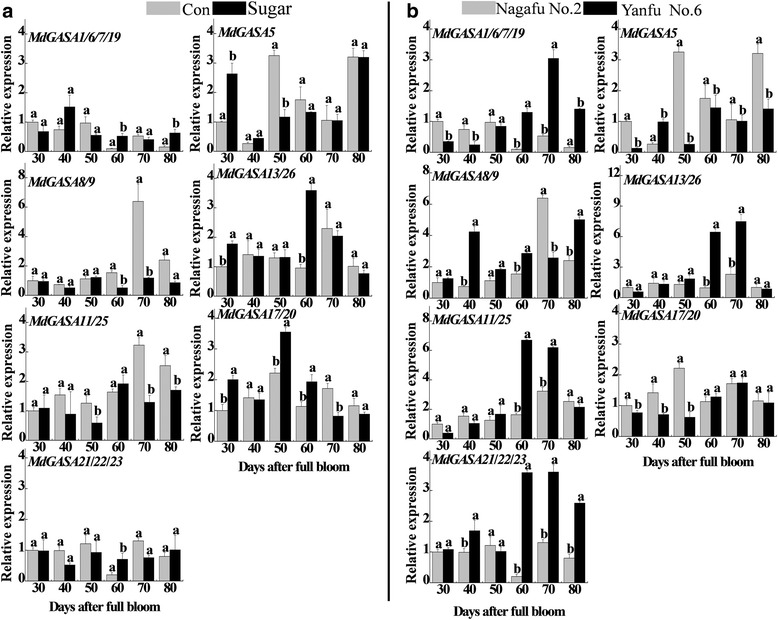
Analysis of flowering-related *MdGASA* expression levels in response to sugar (**a**) and in different apple varieties (‘Nagafu No. 2’ and ‘Yanfu No. 6’) (**b**). Samples were collected at 30, 40, 50, 60, 70, and 80 days after full bloom (DAFB), with water used as a control. Each value represents the mean ± standard error of three replicates. Means followed by small letters are significantly different at the 0.05 level

We also investigated *MdGASA* expression patterns following sugar treatments during the apple flower induction period. The *MdGASA1*/*6*/*7*/*19* transcription levels fluctuated, and were inhibited at 30, 50, and 70 DAFB, while *MdGASA5* expression was inhibited except at 30 and 40 DAFB. Additionally, *MdGASA8/9* expression was down-regulated except at 50 DAFB. Meanwhile, *MdGASA13*/*26* expression levels increased only at 30 and 60 DAFB. Similarly, *MdGASA11*/*25* expression was down-regulated except at 30 and 60 DAFB. Expression-level differences were also detected between *MdGASA17*/*20* and *MdGASA21*/*22*/*23* (Fig. 10a). The considerable increases or decreases in *MdGASA* expression levels in response to sugar treatments implied these genes may be associated with sugar signaling pathways during the flower induction period.

We further analyzed the *MdGASA* expression levels in apple varieties that differed in terms of flowering (‘Nagafu No. 2’ and ‘Yanfu No. 6’) (Fig. 10b). *MdGASA1*/*6*/*7*/*19* expression was down-regulated in ‘Yanfu No. 6’ at the three early sampling dates, but was then up-regulated. Moreover, the *MdGASA5* expression level was lower in ‘Yanfu No. 6’ except at 40 DAFB, while *MdGASA8/9* expression was higher in ‘Yanfu No. 6’ except at 70 DAFB. The expression levels of *MdGASA13*/*26* and *MdGASA11*/*25* were higher in ‘Yanfu No. 6’ only at 60 and 70 DAFB. The transcription level of *MdGASA17*/*20* was lower in ‘Yanfu No. 6’ only at the first three time points, while *MdGASA21*/*22*/*23* expression was higher in ‘Yanfu No. 6’ only at the final three time points.

### Analysis of the *cis*-elements in the *MdGASA* promoters

**Fig. 11:**
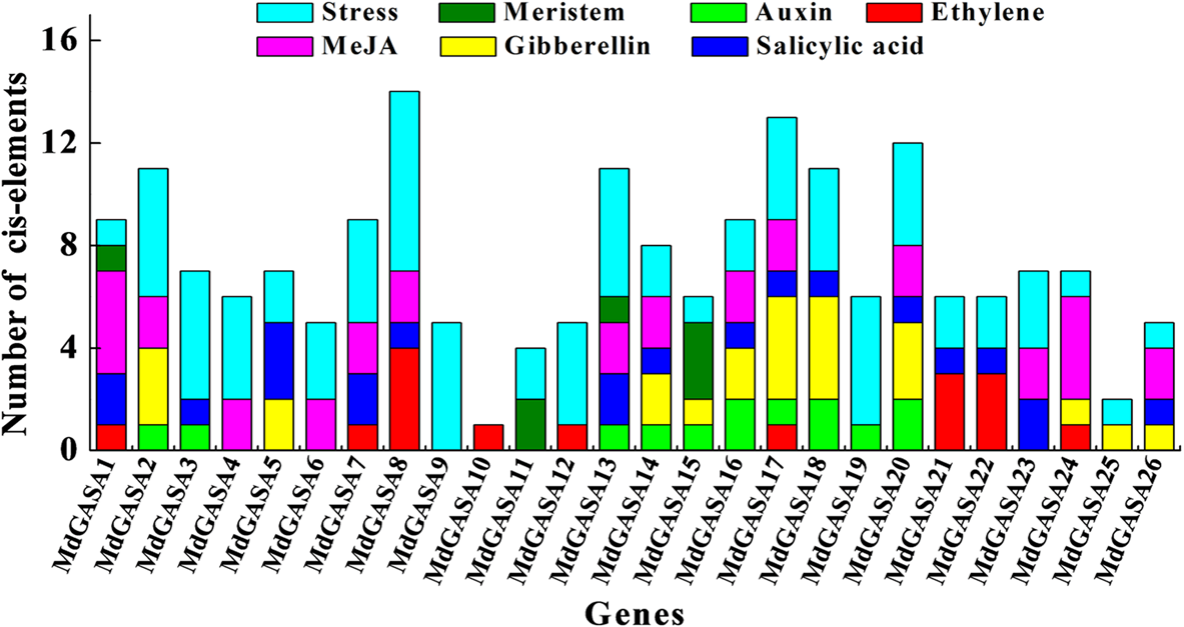
Predicted *cis*-elements in the *MdGASA* promoters. The 1.5-kb sequences of 26 *MdGASA* genes were analyzed with the PlantCARE program

To investigate the regulatory mechanisms of *MdGASA* genes, a 1.5-kb promoter region upstream of the start codon (ATG) was isolated based on the apple genome sequence (Fig. 11) and analyzed to identify potential *cis*-elements [24]. Several stress-related *cis*-elements were detected in the promoters of the 26 candidate *MdGASA* genes. Moreover, meristem-related *cis*-elements were also identified in the *MdGASA1*, *MdGASA11*, *MdGASA13*, and *MdGASA15* promoters. Additional hormone-related *cis*-elements were detected in various *MdGASA* genes*.* These identified motifs indicated *MdGASA* genes might be regulated by *cis*-elements within the corresponding promoters.

## Discussion

The low-molecular weight GASA proteins influence various biological processes and are important for plant growth and development. To our best understanding, a systematic identification of *GASA* genes has only been reported for *Solanum tuberosum* [5]. Little is known about the corresponding apple genes. In this study, we compiled an improved list of candidate *A. thaliana GASA* genes based on a published study [20]. We also identified the candidate apple *GASA* genes, and subsequently characterized the genes in terms of phylogenetic relationships, structure, synteny, and tissue-specific expression patterns. Finally, an analysis of *MdGASA* expression levels in response to various flowering-related factors indicated these genes may affect apple flower induction. This study represents the first comprehensive investigation of the *A. thaliana* and apple *GASA* gene families, and the resulting data will undoubtedly be useful for future analyses, including investigations on the potential roles for GASA proteins during hormone- or sugar-mediated flower induction in fruit trees.

### Genome-wide identification and characterization of *GASA* genes in *Arabidopsis* and apple

We identified 15 and 26 *GASA* genes in the *A. thaliana* and apple genomes, respectively. The fact we detected more *AtGASA* genes than a previous study [20] may have been because we used an updated TAIR database. Additionally, the number of *MdGASA* genes was greater than the number of *A. thaliana* and potato *GASA* genes [5]. A subsequent analysis of the identified *AtGASA* and *MdGASA* genes confirmed they all encoded a conserved GASA domain containing 12 cysteine residues (Fig. 1, Additional file 1) [7–9].

We compared the *A. thaliana* and apple GASA proteins in terms of several characteristics, including length, molecular weight, isoelectric point, instability index, GRAVY, amino acid content, and aliphatic index. Interestingly, all of the identified *GASA* genes were shorter with a lower molecular weight than the members of other known flowering-related gene families (i.e., *SPL*, *MADS*-*box*, and *IDD*) [24, 26, 27, 30]. This observation is consistent with the fact *GASA* genes encode low-molecular weight proteins [2, 5, 31]. Additionally, the amino acid contents of the identified *A. thaliana* and apple GASA proteins were very similar (Table 2). The abundance of Cys residues among the GASA proteins was likely due to the highly conserved 12 cysteines (Fig. 1, Additional file 1). However, we observed differences in the instability index and GRAVY values between the AtGASA and MdGASA proteins, which may be associated with the variability in the intermediate regions (i.e., 7–31 polar amino acids) [5]. Furthermore, all of the identified MdGASA proteins shared similar structures and motifs (e.g., motif 1) (Fig. 4b). Additionally, genes from the same group shared similar exon-intron structures, indicating that the evolution of the GASA domain was associated with genetic structures. However, we detected some structural differences among the *GASA* genes, and the gain or loss of exons or introns may have been a consequence of chromosomal rearrangements [32, 33].

All of the identified *AtGASA* genes were equally distributed on five *A. thaliana* chromosomes (Fig. 5b), while the 26 *MdGASA* genes were located on only 11 of 17 apple chromosomes. Six apple chromosomes (chromosomes 1, 2, 6, 7, 10 and 11) did not contain any copy of the *MdGASA* genes. A similarly uneven distribution of *GASA* genes was reported for potato [5].

### Evolutionary and syntenic relationships among *GASA* genes

Phylogenetic and syntenic relationships among *GASA* genes were analyzed. First, we constructed a phylogenetic tree based on the *A. thaliana* and apple GASA protein sequences (Fig. 3). The 41 GASA protein sequences were clustered into three groups, which were consistent with the phylogenetic classification of potato homologs [5]. The *GASA* genes were nearly equally distributed among the three groups. However, *MdGASA10* did not cluster with the other *GASA* genes according to the phylogenetic tree. This may have been because of the insertion of several amino acids in the GASA domain (Fig. 2). Additionally, the *AtGASA* and *MdGASA* genes were closely clustered, possibly because *A. thaliana* and apple are both dicotyledonous species that may have a common ancestor.

A previous study revealed that tandem, segmental, and whole genome duplications are important for the evolution of species [32]. To date, some apple gene duplications have been characterized (e.g., *SPL*, *IDD*, and *bZIP* genes) [24, 26, 34]. In the current study, we investigated the duplication of *MdGASA* genes. Two gene pairs (*MdGASA7–MdGASA19* and *MdGASA9–MdGASA22*) were likely the result of segmental duplications (Fig. 5a). Additionally, these duplicated genes clustered together in the same phylogenetic group and their expression patterns were suggested to be relatively stable [35]. An earlier study concluded that a recent genome duplication event promoted the expansion of the apple genome, leading to an increase in the number of chromosomes from nine to 17 [28]. Thus, genome duplications have played an important role in the expansion of *MdGASA* genes. Moreover, this gene duplication and expansion may have contributed to an increase in the diversity of *MdGASA* genes regarding quality, structure and function.

Genomic comparisons in which a newly identified gene is compared with orthologous genes from well-characterized plant species are useful for generating information and providing valuable clues regarding gene structures and functions [36–38]. In the current study, we developed a method to analyze syntenic relationships between the apple genome and the well-characterized *A. thaliana* genome to elucidate evolutionary relationships and possible roles of *MdGASA* genes (Fig. 5b). Although potential roles for genes can be ascribed based on the known functions of several well-characterized homologs, further research will be needed to confirm the putative protein functions.

### *MdGASA* gene expression profiles and potential functions

We investigated the expression patterns of *MdGASA* genes in different apple varieties and tissues based on the ArrayExpress database and qRT-PCR. The Group 2 genes (*MdGASA4*, *MdGASA19*, *MdGASA7*, *MdGASA1* and *MdGASA6*) shared similar expression patterns, which was consistent with the similarities in their gene structures (Fig. 4a). In contrast, the genes from Groups 1 and 3 exhibited diverse expression patterns among various tissues. Interestingly, the expression levels of almost all of the identified *MdGASA* genes were down-regulated in seeds or seedlings (Fig. 6). Furthermore, *MdGASA2*, *MdGASA4*, *MdGASA9*, and *MdGASA25* were most highly expressed in leaves, implying they may be important for leaf development [38, 39]. Meanwhile, *MdGASA19*, *MdGASA21*, *MdGASA22*, and *MdGASA23* expression levels were highest in harvested fruits, suggesting their importance in developing fruits [23, 40]. Overall, the various expression patterns were consistent with the differences in gene chromosomal locations, characteristics, and structures.

We further analyzed the *MdGASA* expression patterns among different ‘Nagafu No. 2’ tissues (Fig. 7). Because of the similarities in the *GASA* genes and the fact their coding regions were very short, distinguishing between genes was difficult. Thus, only 17 primer pairs were designed for expression analyses. *MdGASA* genes (except *MdGASA3* and *MdGASA13*/*26*) were highly expressed in the leaves, buds, and flowers, which suggested they play key roles in the development of these tissues [7, 20, 21]. Additionally, in floral biology research, the leaves and buds have traditionally been the important tissues used for analyses of flower induction [41, 42]. Additionally, *MdGASA3* and *MdGASA13*/*26* expression levels were highest in ‘Nagafu No. 2’ fruits, implying these genes have important effects related to fruit development [23, 40]. However, these roles will need to be verified in future studies.

The *GASA* proteins are important for regulating flower induction in various species such as *A. thaliana* [12, 20, 38], rice [4], strawberry [43], *G. hybrida* [21, 22], and *P. hybrida* [3]. However, little is known about their potential roles in regulating flower induction in apple. Consequently, we first investigated *MdGASA* expression patterns to assess whether they are associated with hormone- or sugar-mediated flower induction (Figs 8 and 9). Previous studies confirmed that hormones and sugars have important functions during the flower induction period, and that the application of exogenous GA_3_ decreases the flowering rates of apple trees, while sugar and 6-BA have the opposite effect [24, 44, 45]. We observed that our exogenous GA_3_ treatment almost inhibited the expression of the flowering-related *MdGASA* genes (*MdGASA8*, *MdGASA13*/*26*, *MdGASA11*/*25*, *MdGASA17*/*20*, and *MdGASA21*/*22*/*23*), which is consistent with the previously reported low flowering rate of GA_3_-treated trees [24, 45]. However, the *MdGASA1*/*6*/*7*/*19* expression patterns differed from those of other flowering-related *MdGASA* genes in response to a GA_3_ treatment. The differences in expression patterns might be due to the redundant functions of *GASA* gene family members. For example, over-expressing *GASA5* reportedly delays flowering, while over-expressing *GASA6* leads to an early-flowering phenotype [12, 38]. Overall, our findings indicate that *GASA* genes are involved in GA-mediated flower induction.

Researchers have demonstrated that 6-BA positively affects apple flower induction [44, 46–48]. Several identified gene families are also involved in regulating flower induction in response to exogenous 6-BA treatments [24]. Sugar, as an energy source, is also important for initiating floral development activities [29, 42]. Sugar can be integrated by various flowering pathways to regulate flowering in apple [29]. However, whether *GASA* genes are associated with the relevant 6-BA or sugar signaling pathways has not been addressed. In the current study, we first analyzed the responses of the candidate flowering-related *MdGASA* genes to 6-BA or sugar treatments during the flower induction period (Figs 8b and 9a). The expression levels of most candidate *MdGASA* genes were up- or down-regulated by exogenous applications of 6-BA or sugar, indicating they may influence the regulation of flower induction by 6-BA or sugar. However, because of a relative lack of research on the effects of GASA proteins on 6-BA or sugar signaling, the hypothesis that GASA proteins influence the activities of 6-BA or sugar related to flower induction cannot be verified. Therefore, future research should focus on the potential relationships between MdGASA proteins and 6-BA or sugar signaling. Nevertheless, the results presented herein may be useful for summarizing the potential roles for MdGASA proteins in response to 6-BA and/or sugar.

The *MdGASA* genes exhibited different expression patterns in the ‘Nagafu No. 2’ and ‘Yanfu No. 6’ apple cultivars, which differ in terms of flowering. *MdGASA1/6/7/9* and *MdGASA11/25* exhibited the opposite expression profiles during the first three and final three analyzed time points of the floral induction stage. These observations were consistent with their expression patterns in response to exogenous GA_3_. Thus, these two apple genes likely have opposing functions regarding the regulation of flower induction, similar to *AtGASA5* and *AtGASA6* [12, 38]. However, this will need to be experimentally confirmed. Most of the *GASA* expression levels initially increased and then decreased in ‘Nagafu No. 2’ and ‘Yanfu No. 6’ trees. This expression pattern might be associated with differences in endogenous hormone levels, as ‘Nagafu No. 2’ trees reportedly require a greater abundance of hormones for growth [49]. To further investigate whether the expression of *MdGASA* genes is regulated by different hormones or signals, the *cis*-elements in the corresponding promoters were analyzed as previously described [24]. We observed that most of the *GASA* promoters had common hormone- and stress-related *cis*-elements, which may be involved in the complex regulatory mechanisms affecting gene expression (Fig. 10).

## Conclusion

We identified 15 *AtGASA* genes and 26 *MdGASA* genes. All of the identified *GASA* genes included a conserved *GASA* domain, and exhibited similar physical and chemical characteristics. A phylogenetic analysis revealed that *AtGASA* and *MdGASA* genes can be classified into three groups. Additional analyses of synteny and gene duplications helped to elucidate the expansion and diversity of *MdGASA* genes. Moreover, an analysis of the spatiotemporal tissue-specific expression patterns indicated that most *MdGASA* genes were expressed more in the leaves, buds, and fruits than in the seeds and seedlings. Additionally, the expression levels of selected candidate flowering-related *MdGASA* genes were further analyzed in different varieties and in response to different treatments (i.e., GA_3_, 6-BA, and sugar). The resulting data indicated the *MdGASA* genes may help to regulate the induction of flowering in apple trees. Overall, our comprehensive genome-level investigation of *A. thaliana* and apple *GASA* genes provides basic relevant information for future studies. The data presented herein may help to support hypotheses regarding the involvement of GASA during the flower induction stage of fruit tree species.

## Methods

### Identification of *Arabidopsis thaliana* and apple *GASA* genes

AtGASA were identified from the *Arabidopsis* database (http://www.arabidopsis.org). Another two new *GASA* genes (AT3g10185 and AT1g10588) were replenished based on a previous study [20]. They were named *AtGASA14* and *AtGASA15*.To identify apple *GASA* genes, we first used the 15 identified AtGASA protein sequences as queries to search the apple genome database (*Malus domestica* Genome v1.0, http://www.rosaceae.org/) (Additional file 7). The obtained sequences were then used as queries to search the conserved domain database (https://www.ncbi.nlm.nih.gov/Structure/cdd/wrpsb.cgi). and to discard genes lacking a *GASA* domain. All non-redundant putative protein sequences were finally manually checked to confirm the presence of the GASA domain.

### Chemical characterization, chromosome mapping and sequence alignments

The sequences of the candidate *A. thaliana* and apple GASA proteins were used to predict protein characteristics with the ExPASy program (http://web.expasy.org/protparam/). Additionally, the physical location of each *MdGASA* gene was determined according to the genome annotations. The genes were then mapped to specific chromosomes. The DNAMAN program was used to align the MdGASA and AtGASA protein sequences, while the WebLogo platform (http://weblogo.berkeley.edu/logo.cgi) was used to generate and analyze sequence logos. The TMHMM server v2.0 (http://www.cbs.dtu.dk/services/TMHMM/) was used to predict the presence of transmembrane helices, while the predicted MdGASA protein structures were analyzed with the PHYRE server v2.0 (http://www.sbg.bio.ic.ac.uk/phyre2/html/page.cgi?id=index).

### Analyses of phylogenetic relationships, gene structures and motifs, and promoters

A phylogenetic tree comprising all candidate *A. thaliana* and apple *GASA* genes was constructed using the neighbor-joining method of the MEGA 6.06 program. Default parameters were used with a bootstrap test involving 1000 replicates. The Gene Structure Display Server (http://gsds.cbi.pku.edu.cn/) was used to construct exon-intron structures. The gene structures were determined based on the coding sequences within the corresponding genomic sequence. The MEME platform (http://meme-suite.org/) was used to identify the conserved motifs in the MdGASA proteins [50] (default parameters with the maximum number of motifs set to 10). Furthermore, the 1.5-kb region upstream of the start codon of candidate *MdGASA* genes was examined for the presence of *cis*-elements. The PlantCARE program (http://bioinformatics.psb.ugent.be/webtools/plantcare/html/) was used to search for regulatory elements.

### Plant materials and treatments

Two-year-old pot-cultivated ‘Nagafu No. 2’ apple trees grown on M.26 rootstocks underwent various treatments. Hormone treatments involved the application of 100 mΜ GA_3_ or 300 μM ABA to apple leaves, which were then collected at 0, 1, 3, 6, and 12 h after treatment.

Seventy-two uniformly growing 6-year-old ‘Fuji’/T337/*Malus robusta* Rehd. apple trees were randomly divided into four groups of 18 trees each. The groups were then treated with GA_3_, sucrose, 6-BA, or water (control). The trees were then grown at an experimental orchard in Yangling, China (108°04′ E, 34°16′ N). Each group was prepared as three blocks, with three replicates. The trees were analyzed from 30 to 80 DAFB in 2015. The GA_3_ treatment was completed using a slightly modified previously described method [45]. Briefly, 700 mg L^−1^ GA_3_ (Sigma, Deisenhofen, Germany) was sprayed once on a clear morning at 30 DAFB (May 9). Additionally, trees were sprayed with 300 mg L^−1^ 6-BA (Sigma) on a clear morning at 30 DAFB (May 9). The sugar treatment involved spraying trees two times with 15,000 mg L^−1^ and 20,000 mg L^−1^ sucrose on clear mornings at 30 and 37 DAFB (May 9 and May 16). All treatments involved the whole tree and were applied with a low-pressure hand-wand sprayer. Terminal buds on the current-year spurs (< 5 cm), which were chosen according to previous studies [24, 25, 29], were collected at 30, 40, 50, 60, 70, and 80 DAFB and immediately frozen with liquid nitrogen and stored at −80 °C until used in gene expression analyses.

Buds from two apple varieties (‘Yanfu No. 6’ and ‘Nagafu No. 2’) were collected from 18 uniformly growing 6-year-old trees in 2015 at 30, 40, 50, 60, 70, and 80 DAFB. ‘Yanfu No. 6’ is a spontaneous mutant of ‘Nagafu No. 2’ that produces more flower buds. Moreover, ‘Yanfu No. 6’ trees produce a higher proportion of spurs, shorter internodes, bigger buds, and more flowers. Terminal buds on the current-year spurs (< 5 cm) were collected as described above.

Different organs were also collected for analyses of tissue-specific expression patterns. Flowers were collected at full bloom on April 9 in 2015. Additionally, stems were collected from new shoots with a diameter of 2–3 mm, while mature leaves were collected from the adjacent buds. Fruits with a diameter of 2–3 cm were also collected. All samples were immediately frozen in liquid nitrogen and stored at −80 °C until used in gene expression analyses.

### RNA extraction and cDNA synthesis

Total RNA was extracted from plant tissue samples using a slightly modified cetyltrimethylammonium bromide (CTAB) method [51]. Briefly, 900 μL extraction buffer (2% CTAB, 2.5% PVP-40, 2 M NaCl, 100 mM Tris-HCl [pH 8.0], 25 mM EDTA [pH 8.0], and 2% β-mercaptoethanol) was pre-heated at 65 °C and added to 2-mL microcentrifuge tubes just before use. Bud samples (200 mg) stored at −80 °C were ground to a powder and then added to the extraction buffer in microcentrifuge tubes. After vigorously shaking and inverting each tube for 5 min and incubating at 65 °C for 30 min, an equal volume of chloroform:isoamyl alcohol (24:1, *v*/v) was added. The tube was vigorously shaken and inverted and then centrifuged at 12,000×*g* for 10 min at 4 °C. The supernatant was transferred to a new tube and re-extracted with an equal volume of chloroform:isoamyl alcohol (24:1, v/v). The supernatant was transferred to a new 2-mL tube, after which LiCl (3 M final concentration) was added. The mixture was incubated at −20 °C for 4 h, after which the RNA was selectively pelleted by LiCl after a centrifugation at 18,000×*g* for 20 min at 4 °C. The pellet was resuspended in 500 μL SSTE buffer (10 mM Tris-HCl [pH 8.0], 1 mM EDTA [pH 8.0], 1% SDS, and 1 M NaCl) that had been pre-heated to 65 °C and an equal volume of chloroform:isoamyl alcohol. The mixture was then centrifuged at 12,000×g for 10 min at 4 °C. The supernatant was transferred to a new microcentrifuge tube, and the RNA was precipitated with 2.5 volumes of cold ethanol at −80 °C for at least 30 min. After a centrifugation at 12,000×g for 20 min at 4 °C, the pellets were washed with 70% ethanol and resuspended in diethylpyrocarbonate-treated water. Total RNA integrity was verified by 2% agarose gel electrophoresis. Additionally, first-strand cDNA was synthesized from 1 μg total RNA using a PrimeScript RT Reagent kit with gDNA Eraser (Takara Bio, Shiga, Japan).

### Analysis of *GASA* expression

The expression patterns of candidate *MdGASA* genes were analyzed by qRT-PCR. All primer pairs were designed with the Primer 5.0 program (Additional file 8). However, because the coding sequences of the *MdGASA* genes were very short and highly conserved, we were unable to specifically amplify each gene. Thus, only 17 primer pairs were designed to analyze all 27 *MdGASA* genes. Two or three genes were amplified using previously designed primers [24, 26]. Consequently, *MdGASA1*, *MdGASA6*, *MdGASA7*, and *MdGASA19* were amplified by the same primer pair. Additionally, *MdGASA8* and *MdGASA9*, *MdGASA11* and *MdGASA25*, *MdGASA13* and *MdGASA26*, *MdGASA17* and *MdGASA20* were amplified by the same primer pairs, respectively. Finally, *MdGASA21*, *MdGASA22*, and *MdGASA23* were also amplified by the same primers.

The qRT-PCR assay mix (20 μL) consisted of 2 μL cDNA (diluted 1:8), 10 μL 2× SYBR Premix ExTaq II (Takara Bio), 0.8 μL each primer (10 μM) (Table 1), and 6.4 μL distilled deionized H_2_O. Each qRT-PCR assay was completed on an iCycler iQ Real Time PCR Detection System (Bio-Rad) with the following program: 95 °C for 3 min; 40 cycles at 94 °C for 15 s, 62 °C for 20 s, and 72 °C for 20 s. The resulting fragments were immediately subjected to a melting-curve analysis to verify the presence of gene-specific PCR products. The melting-curve analysis was completed with the following program: 94 °C for 15 s, followed by a constant increase from 60 °C to 95 °C at a 2% ramping rate. The apple *EF-1α* gene (GenBank accession No. DQ341381) was used as an internal control and served as the standard for normalizing all mRNA levels. The 2^−ΔΔCt^ method was used to calculate the relative template abundance in each PCR amplification mixture [52]. Three biological replicates were used for gene expression analysis [53].

**Table 1:** *Arabidopsis thaliana* and apple *GASA* gene families

Gene Name	Gene Locus^a^	Location	CDS (bp)	Peptide (aa)	Molecular weight(KD)
*AtGASA1*	AT1G75750	chr1:28,441,526..28,442,367	297	98	10.74
*AtGASA2*	AT4G09610	chr4:6,074,770..6,075,645	300	99	10.53
*AtGASA3*	AT4G09600	chr4:6,072,804..6,073,612	300	99	10.70
*AtGASA4*	AT5G15230	chr5:4,944,900..4,946,216	322	106	12.00
*AtGASA5*	AT3G02885	chr3:638,021..639,055	294	97	10.85
*AtGASA6*	AT1G74670	chr1:28,053,286..28,054,149	306	101	11.34
*AtGASA7*	AT2G14900	chr2:6,404,175..6,405,330	327	108	11.38
*AtGASA8*	AT2G39540	chr2:16,500,866..16,501,241	264	87	9.44
*AtGASA9*	AT1G22690	chr1:8,027,294..8,028,125	360	119	12.94
*AtGASA10*	AT5G59845	chr5:24,111,324..24,112,020	270	89	9.75
*AtGASA11*	AT2G18420	chr2:7,993,801..7,994,554	285	94	10.15
*AtGASA12*	AT2G30810	chr2:13,127,826..13,128,666	321	106	11.67
*AtGASA13*	AT5G14920	chr5:4,826,479..4,827,980	828	275	29.14
*AtGASA14*	AT1G10588	chr1:3,501,202..3,501,904	273	90	9.81
*AtGASA15*	AT3G10185	chr3:3,145,579..3,146,199	312	103	11.37
*MdGASA1*	MDP0000297328	chr3:6,467,601..6,468,215	267	88	9.71
*MdGASA2*	MDP0000338377	chr3:20,700,888..20,702,137	345	116	12.76
*MdGASA3*	MDP0000144384	chr4:14,956,772..14,957,278	312	103	11.48
*MdGASA4*	MDP0000201700	chr5:25,382,370..25,382,887	267	88	9.74
*MdGASA5*	MDP0000937996	chr8:953,084..953,627	315	105	11.06
*MdGASA6*	MDP0000366256	chr8:12,351,688..12,352,326	339	114	12.86
*MdGASA7*	MDP0000150771	chr8:12,404,423..12,405,011	267	88	9.69
*MdGASA8*	MDP0000269551	chr9:2,230,511..2,231,810	765	254	27.12
*MdGASA9*	MDP0000157876	chr9:2,230,957..2,231,851	600	199	21.36
*MdGASA10*	MDP0000164286	chr9:2,239,485..2,240,306	465	154	17.58
*MdGASA11*	MDP0000229958	chr9:3,175,216..3,176,189	327	108	11.90
*MdGASA12*	MDP0000150141	chr9:15,545,432..15,546,460	348	115	12.65
*MdGASA13*	MDP0000212045	chr9:29,285,476..29,286,214	285	94	10.44
*MdGASA14*	MDP0000140075	chr12:21,213,945..21,214,429	321	106	11.69
*MdGASA15*	MDP0000251418	chr12:21,215,744..21,216,304	324	107	11.32
*MdGASA16*	MDP0000251419	chr12:21,217,841..21,218,322	306	101	11.16
*MdGASA17*	MDP0000786380	chr13:5,529,785..5,530,873	327	109	11.88
*MdGASA18*	MDP0000126347	chr14:12,628,198..12,630,144	351	116	12.85
*MdGASA19*	MDP0000195254	chr15:1,842,484..1,843,065	267	88	9.71
*MdGASA20*	MDP0000230952	chr16:3,972,710..3,974,022	525	174	19.43
*MdGASA21*	MDP0000735118	chr17:2,662,778..2,663,828	528	175	19.04
*MdGASA22*	MDP0000901967	chr17:2,663,997..2,665,047	528	175	24.71
*MdGASA23*	MDP0000126601	chr17:2,665,111..2,666,161	528	175	20.07
*MdGASA24*	MDP0000663790	chr17:2,692,170..2,694,350	915	305	33.97
*MdGASA25*	MDP0000232908	chr17:3,533,847..3,534,966	327	108	11.85
*MdGASA26*	MDP0000209689	chr17:22,513,908..22,514,654	285	94	8.63

**Table 2:** Amino acid compositions as well as physical and chemical characteristics of GASA proteins

Proteins	Isoelectric Point	Instability Index	GRAVY^a^	Major Amino Acid^b^	Aliphatic Index
*AtGASA1*	9.40	43.38	−0.155	C(12.2%)L(10.2%)AR(9.2%)	79.69
*AtGASA2*	8.98	32.78	0.175	C(13.1%)L(10.1%)STV(8.1%)	84.65
*AtGASA3*	8.88	45.34	0.154	C(13.1%)L(11.1%)R(8.1%)	82.73
*AtGASA4*	9.46	57.75	−0.379	CM(11.3%)GS(7.5%)L(6.6%)	54.25
*AtGASA5*	9.68	38.56	−0.192	C(13.4%)K(12.4%)L(10.3%)	58.35
*AtGASA6*	9.01	45.71	−0.251	C(12.9%)K(%)GLPT(7.9%)	50.20
*AtGASA7*	8.74	33.70	−0.060	CKS(11.1%)AG(9.3%)L(8.3%)	74.17
*AtGASA8*	8.63	47.84	−0.082	CS(13.8%)K(10.3%)G(8.0%)	62.64
*AtGASA9*	9.52	46.47	−0.334	S(12.6%)C(10.1%)A(8.4%)	61.51
*AtGASA10*	8.97	42.68	−0.208	C(13.5%)KS(11.2%)L(6.7%)	64.61
*AtGASA11*	8.67	35.87	0.013	C(12.8%)L(10.6%)S(9.6%)	75.74
*AtGASA12*	7.96	47.57	−0.371	C(11.3%)K(9.4%)E(8.5%)	53.40
*AtGASA13*	9.98	64.07	−0.525	P(29.5%)t(14.9%)v(9.5%)	51.96
*AtGASA14*	7.41	58.35	−0.196	CS(13.3%K(8.9%))DGV(6.7%)	57.33
*AtGASA15*	8.89	33.20	0.096	C(13.6%)LK(9.7%)P(8.7%)	77.57
*MdGASA1*	8.42	34.06	−0.191	C(14.8%)K(10.2%)S(10.2%)	52.05
*MdGASA2*	9.30	40.54	−0.241	C(11.2%)P(8.6%)L(8.6%)	62.24
*MdGASA3*	7.97	54.52	−0.207	C(11.7%)A(8.7%)S(7.8%)	55.92
*MdGASA4*	8.70	35.18	−0.170	C(14.8%)K(11.4%)P L(8.0%)	52.16
*MdGASA5*	8.93	48.40	0.030	C(11.5%)L(11.5%)S(10.6%)	82.50
*MdGASA6*	8.39	40.83	0.039	C(12.3%)L(10.5%)K(9.6%)	62.46
*MdGASA7*	8.59	36	−0.024	C(14.8%)K(10.2%)S(9.1%)	52.05
*MdGASA8*	10.05	77.52	−0.467	P(30.3%)K(9.8%)T(9.1%)	65.28
*MdGASA9*	9.85	78.12	−0.666	P(30.7%)T(11.1%)K(8.5%)	49.95
*MdGASA10*	9.09	50.24	−0.467	K(10.4%)P(10.4%)C S(7.8%)	63.38
*MdGASA11*	9.36	47.54	−0.247	C(11.1%)K G(9.3) A Q(8.3%)	41.67
*MdGASA12*	8.97	40.55	−0.237	A(11.3%)C(10.4%)K(10.4%)	59.57
*MdGASA13*	9.28	47.42	−0.104	C(12.8%)K(10.6%)P(8.5%)	62.23
*MdGASA14*	8.93	47.14	0.015	C(11.3%)R(8.5%)L(8.5%)	74.53
*MdGASA15*	9.04	50.16	0.024	C(11.2%)A L S(10.3%)	80.28
*MdGASA16*	8.75	48.77	0.193	C(12.9%)P(8.9%)L T V(6.9%)	72.38
*MdGASA17*	8.94	49.49	−0.148	C(11.1%)R(9.3%)L T V(8.3%)	70.37
*MdGASA18*	9.00	47.84	−0.345	C(10.3%)P(9.5%)N T(8.6%)	47.16
*MdGASA19*	8.42	41.20	−0.108	C(14.8%)K(11.4%)S(10.2%)	52.05
*MdGASA20*	8.98	44.92	−0.348	LT(8.6%)C S(8.0%)A R V(7.5%)	69.48
*MdGASA21*	9.67	74.12	−0.306	P(21.7%)K(8.6%)L V(8.0%)	65.71
*MdGASA22*	4.37	60.15	−0.906	D(14.8%)S(12.6%)A(8.3%)	50.13
*MdGASA23*	4.11	53.65	−0.837	D(15.7%)S(11.9%)G(7.0%)	50.65
*MdGASA24*	10.14	65.09	−0.683	P(16.4%)K(11.5%)S(10.9%)	60.92
*MdGASA25*	9.27	51.16	−0.218	C(11.1%)G K(9.3%)A(8.3%)	48.89
*MdGASA26*	9.20	52.53	0.253	C(13.8%)L(11.2%)K S(10.0%)	74.38

^a^Grand average of hydropathicity

^b^The three main amino acids for each protein

(*A* Ala, *P* Pro, *S* Ser, *G* Gly, *L* Leu, *N* ASN, *K* Lys, *C* Cys, *V* Val, *R* Arg, *P* Pro, *Q* Gln, *M* Met, *T* Thr)

### Statistical analysis

Data underwent an analysis of variance and the means were compared by a *t*-test at the 5% level using the SPSS 11.5 software package (SPSS, Chicago, IL, USA). Figures were prepared using Origin 7.5 (Microcal Software Inc., Northampton, MA, USA).

## Additional files

Additional file 1: Alignment of GASA domains from AtGASA proteins. (a) Multiple alignments of the AtGASA protein sequences and their conserved GASA domains, red column represented their conserved twelve cysteines. (b) Sequence logo analysis of the conserved AtGASA domains. Each stack represented their amino acids. (TIFF 5642 kb)

Additional file 2: Transmembrane topology analysis of MdGASA proteins. Transmembrane helices of the MdGASA proteins were predicted with the TMHMM server v2.0. The red peaks indicate the predicted transmembrane helices. (TIFF 3853 kb)

Additional file 3: Secondary structures of MdGASA protein. Their α helix, Extended strand, Random coil and β turn were analyzed. (DOCX 14 kb)

Additional file 4: Motif sequence identified by MEME. Motif number was associated with fig. 4c. (TIFF 7247 kb)

Additional file 5: Effects of GA_3_ and ABA on leaf *MdGASA* expression levels. Leaves were collected at 0, 1, 3, 6 and 12 h after each treatment. 100Μm GA_3_, and 300 μM ABA were sprayed in apple leaves. (TIFF 6945 kb)

Additional file 6: *MdGASA* expression levels based on previous RNA sequencing data. (XLSX 10 kb)

Additional file 7: Details regarding identified *Arabidopsis thaliana* and apple *GASA* genes. Gene names and protein sequences were listed. (XLSX 14 kb)

Additional file 8: Sequences of primers used to amplify *MdGASA* genes and their reference genes. (DOCX 13 kb)

## Abbreviations

6-BA: 6-benzylaminopurine; ABA: abscisic acid
DAFB: Days after full blossom
GA_3_: Gibberellic acid
qRT-PCR: Quantitative real-time polymerase chain reaction

## References

[1] Shi LF, Gast RT, Gopalraj M, Olszewski NE (1992). Characterization of a shoot-specific, Ga3-regulated and Aba-regulated gene from tomato. Plant J.

[2] Herzog M, Dorne AM, Grellet F (1995). GASA, a gibberellin-regulated gene family from Arabidopsis Thaliana related to the tomato GAST1 gene. Plant Mol Biol.

[3] Ben-Nissan G, Lee JY, Borohov A, Weiss D (2004). GIP, a Petunia Hybrida GA-induced cysteine-rich protein: a possible role in shoot elongation and transition to flowering. Plant J.

[4] Furukawa T, Sakaguchi N, Shimada H (2006). Two OsGASR genes, rice GAST homologue genes that are abundant in proliferating tissues, show different expression patterns in developing panicles. Genes Genet Syst.

[5] Nahirnak V, Rivarola M, de Urreta MG, Paniego N, Hopp HE, Almasia NI, Vazquez-Rovere C (2016). Genome-wide analysis of the Snakin/GASA gene family in Solanum Tuberosum cv. Kennebec. Am J Potato Res.

[6] Zhang LY, Geng XL, Zhang HY, Zhou CL, Zhao AJ, Wang F, Zhao Y, Tian XJ, Hu ZR, Xin MM (2017). Isolation and characterization of heat-responsive gene TaGASR1 from wheat (Triticum Aestivum L.). J Plant Biol.

[7] Aubert D, Chevillard M, Dorne AM, Arlaud G, Herzog M (1998). Expression patterns of GASA genes in Arabidopsis Thaliana: the GASA4 gene is up-regulated by gibberellins in meristematic regions. Plant Mol Biol.

[8] Silverstein KA, Moskal WA, Wu HC, Underwood BA, Graham MA, Town CD, VandenBosch KA (2007). Small cysteine-rich peptides resembling antimicrobial peptides have been under-predicted in plants. Plant J.

[9] Zhang SC, Wang XJ (2008). Expression pattern of GASA, downstream genes of DELLA, in Arabidopsis. Chinese Sci Bull.

[10] Rubinovich L, Weiss D (2010). The Arabidopsis cysteine-rich protein GASA4 promotes GA responses and exhibits redox activity in bacteria and in planta. Plant J.

[11] Sun S, Wang H, Yu H, Zhong C, Zhang X, Peng J, Wang X (2013). GASA14 regulates leaf expansion and abiotic stress resistance by modulating reactive oxygen species accumulation. J Exp Bot.

[12] Zhang S, Yang C, Peng J, Sun S, Wang X (2009). GASA5, a regulator of flowering time and stem growth in Arabidopsis Thaliana. Plant Mol Biol.

[13] Wang L, Wang Z, Xu YY, Joo SH, Kim SK, Xue Z, Xu ZH, Wang ZY, Chong K (2009). OsGSR1 is involved in crosstalk between gibberellins and brassinosteroids in rice. Plant J.

[14] Bindschedler LV, Whitelegge JP, Millar DJ, Bolwell GP (2006). A two component chitin-binding protein from French bean - association of a proline-rich protein with a cysteine-rich polypeptide. FEBS Lett.

[15] Mao ZC, Zheng JY, Wang YS, Chen GH, Yang YH, Feng DX, Xie BY (2011). The new CaSn gene belonging to the snakin family induces resistance against root-knot nematode infection in pepper. Phytoparasitica.

[16] Balaji V, Smart CD (2012). Over-expression of snakin-2 and extensin-like protein genes restricts pathogen invasiveness and enhances tolerance to Clavibacter michiganensis subsp michiganensis in transgenic tomato (Solanum Lycopersicum). Transgenic Res.

[17] Wigoda N, Ben-Nissan G, Granot D, Schwartz A, Weiss D (2006). The gibberellin-induced, cysteine-rich protein GIP2 from Petunia Hybrida exhibits in planta antioxidant activity. Plant J.

[18] Li KL, Bai X, Li Y, Cai H, Ji W, Tang LL, Wen YD, Zhu YM (2011). GsGASA1 mediated root growth inhibition in response to chronic cold stress is marked by the accumulation of DELLAs. J Plant Physiol.

[19] Zhang SC, Wang XJ (2011). Overexpression of GASA5 increases the sensitivity of Arabidopsis to heat stress. J Plant Physiol.

[20] Roxrud I, Lid SE, Fletcher JC, Schmidt ED, Opsahl-Sorteberg HG (2007). GASA4, one of the 14-member Arabidopsis GASA family of small polypeptides, regulates flowering and seed development. Plant & cell physiology.

[21] Kotilainen M, Helariutta Y, Mehto M, Pollanen E, Albert VA, Elomaa P, Teeri TH (1999). GEG participates in the regulation of cell and organ shape during corolla and carpel development in Gerbera Hybrida. Plant Cell.

[22] Peng JZ, Lai LJ, Wang XJ (2010). Temporal and spatial expression analysis of PRGL in Gerbera Hybrida. Mol Biol Rep.

[23] Moyano-Canete E, Bellido ML, Garcia-Caparros N, Medina-Puche L, Amil-Ruiz F, Gonzalez-Reyes JA, Caballero JL, Munoz-Blanco J, Blanco-Portales R (2013). FaGAST2, a strawberry ripening-related gene, acts together with FaGAST1 to determine cell size of the fruit receptacle. Plant Cell Physiol.

[24] Fan S, Zhang D, Xing L, Qi S, Du L, Wu H, Shao H, Li Y, Ma J, Han M: Phylogenetic analysis of IDD gene family and characterization of its expression in response to flower induction in Malus. Molecular genetics and genomics. MGG. 2017;292:755–71. 10.1007/s00438-017-1306-4 28314937

[25] Xing LB, Zhang D, Zhao CP, Li YM, Ma JJ, An N, Han MY (2016). Shoot bending promotes flower bud formation by miRNA-mediated regulation in apple (Malus Domestica Borkh.). Plant Biotechnol J.

[26] Li J, Hou H, Li X, Xiang J, Yin X, Gao H, Zheng Y, Bassett CL, Wang X (2013). Genome-wide identification and analysis of the SBP-box family genes in apple (Malus x domestica Borkh.). Plant Physiol Biochem.

[27] Kumar G, Arya P, Gupta K, Randhawa V, Acharya V, Singh AK: Comparative phylogenetic analysis and transcriptional profiling of MADS-box gene family identified DAM and FLC-like genes in apple (Malus x domestica). Sci Rep-Uk. 2016;6:20695. 10.1038/srep20695 PMC4746589 26856238

[28] Velasco R, Zharkikh A, Affourtit J, Dhingra A, Cestaro A, Kalyanaraman A, Fontana P, Bhatnagar SK, Troggio M, Pruss D (2010). The genome of the domesticated apple (Malus x domestica Borkh.). Nat Genet.

[29] Xing LB, Zhang D, Li YM, Shen YW, Zhao CP, Ma JJ, An N, Han MY (2015). Transcription profiles reveal sugar and hormone signaling pathways mediating flower induction in apple (Malus Domestica Borkh.). Plant Cell Physiol.

[30] Tian Y, Dong QL, Ji ZR, Chi FM, Cong PH, Zhou ZS (2015). Genome-wide identification and analysis of the MADS-box gene family in apple. Gene.

[31] Berrocal-Lobo M, Segura A, Moreno M, Lopez G, Garcia-Olmedo F, Molina A (2002). Snakin-2, an antimicrobial peptide from potato whose gene is locally induced by wounding and responds to pathogen infection. Plant Physiol.

[32] Xu GX, Guo CC, Shan HY, Kong HZ (2012). Divergence of duplicate genes in exon-intron structure. P Natl Acad Sci USA.

[33] Guo RR, Xu XZ, Carole B, Li XQ, Gao M, Zheng Y, Wang XP. Genome-wide identification, evolutionary and expression analysis of the aspartic protease gene superfamily in grape. BMC Genomics. 2013;14 10.1186/1471-2164-14-554 PMC3751884 23945092

[34] Zhao J, Guo RR, Guo CL, Hou HM, Wang XP, Gao H. Evolutionary and expression analyses of the apple basic Leucine zipper transcription factor family. Front Plant Sci. 2016;7 10.3389/fpls.2016.00376 PMC4811886 27066030

[35] Zhang JZ (2003). Evolution by gene duplication: an update. Trends Ecol Evol.

[36] Koonin EV (2005). Orthologs, paralogs, and evolutionary genomics. Annu Rev Genet.

[37] Lyons E, Pedersen B, Kane J, Alam M, Ming R, Tang HB, Wang XY, Bowers J, Paterson A, Lisch D (2008). Finding and comparing Syntenic regions among Arabidopsis and the Outgroups papaya, poplar, and grape: CoGe with Rosids. Plant Physiol.

[38] Qu J, Kang SG, Hah C, Jang JC (2016). Molecular and cellular characterization of GA-stimulated transcripts GASA4 and GASA6 in Arabidopsis Thaliana. Plant Sci.

[39] Nahirnak V, Almasia NI, Fernandez PV, Hopp HE, Estevez JM, Carrari F, Vazquez-Rovere C (2012). Potato Snakin-1 gene silencing affects cell division, primary metabolism, and Cell Wall composition. Plant Physiol.

[40] Blanco-Portales R, Bellido ML, Garcia-Caparros N, Medina-Puche L, Caballero-Repullo JL, Gonzalez-Reyes JA, Munoz-Blanco J, Moyano E (2012). The strawberry FaGAST2 gene determines receptacle cell size during fruit development and ripening. FEBS J.

[41] Monerri C, Fortunato-Almeida A, Molina RV, Nebauer SG, Garcia-Luis A, Guardiola JL (2011). Relation of carbohydrate reserves with the forthcoming crop, flower formation and photosynthetic rate, in the alternate bearing ‘Salustiana’ sweet orange (Citrus Sinensis L.). Sci Hortic-Amsterdam.

[42] Fan S, Zhang D, Lei C, Chen HF, Xing LB, Ma JJ, Zhao CP, Han MY (2016). Proteome analyses using iTRAQ labeling reveal critical mechanisms in alternate bearing Malus Prunifolia. J Proteome Res.

[43] de la Fuente JI, Amaya I, Castillejo C, Sanchez-Sevilla JF, Quesada MA, Botella MA, Valpuest V (2006). The strawberry gene FaGAST affects plant growth through inhibition of cell elongation. J Exp Bot.

[44] Li YM, Zhang D, Xing LB, Zhang SW, Zhao CP, Han MY (2016). Effect of exogenous 6-benzylaminopurine (6-BA) on branch type, floral induction and initiation, and related gene expression in ‘Fuji’ apple (Malus Domestica Borkh). Plant Growth Regul.

[45] Zhang S, Zhang D, Fan S, Du L, Shen Y, Xing L, Li Y, Ma J, Han M (2016). Effect of exogenous GA3 and its inhibitor paclobutrazol on floral formation, endogenous hormones, and flowering-associated genes in ‘Fuji’ apple (Malus Domestica Borkh.). Plant Physiol Biochem.

[46] Ito A, Hayama H, Kaskimura Y, Yoshioka H (2001). Effect of maleic hydrazide on endogenous cytokinin contents in lateral buds, and its possible role in flower bud formation on the Japanese pear shoot. Sci Hortic-Amsterdam.

[47] Bernier G, Perilleux C (2005). A physiological overview of the genetics of flowering time control. Plant Biotechnol J.

[48] Krasniqi AL, Damerow L, Kunz A, Blanke MM (2013). Quantifying key parameters as elicitors for alternate fruit bearing in cv. ‘Elstar’ apple trees. Plant Sci.

[49] Song CH, Zhang D, Zheng LW, Zhang J, Zhang BJ, Luo WW, Li YM, Li GF, Ma JJ, Han MY. miRNA and Degradome sequencing reveal miRNA and their target genes that may mediate shoot growth in spur type mutant “Yanfu 6”. Front Plant Sci. 2017;8 10.3389/fpls.2017.00441 PMC5371658 28424721

[50] Bailey TL, Williams N, Misleh C, Li WW (2006). MEME: discovering and analyzing DNA and protein sequence motifs. Nucleic Acids Res.

[51] Gambino G, Perrone I, Gribaudo I (2008). A rapid and effective method for RNA extraction from different tissues of grapevine and other Woody plants. Phytochem Analysis.

[52] Livak KJ, Schmittgen TD (2001). Analysis of relative gene expression data using real-time quantitative PCR and the 2(T)(−Delta Delta C) method. Methods.

[53] Fan S, Zhang D, Gao C, Zhao M, Wu H, Li Y, Shen Y, Han M (2017). Identification, classification, and expression analysis of GRAS gene family in Malus Domestica. Front Physiol.

