# Supplementary figures and images for: Comprehensive analysis of GASA family members in the Malus domestica genome: identification, characterization, and their expressions in response to apple flower induction
Source: BMC Genomics. 2017 Oct 27;18:827. doi: 10.1186/s12864-017-4213-5 (PMC5658915; doi:10.1186/s12864-017-4213-5)

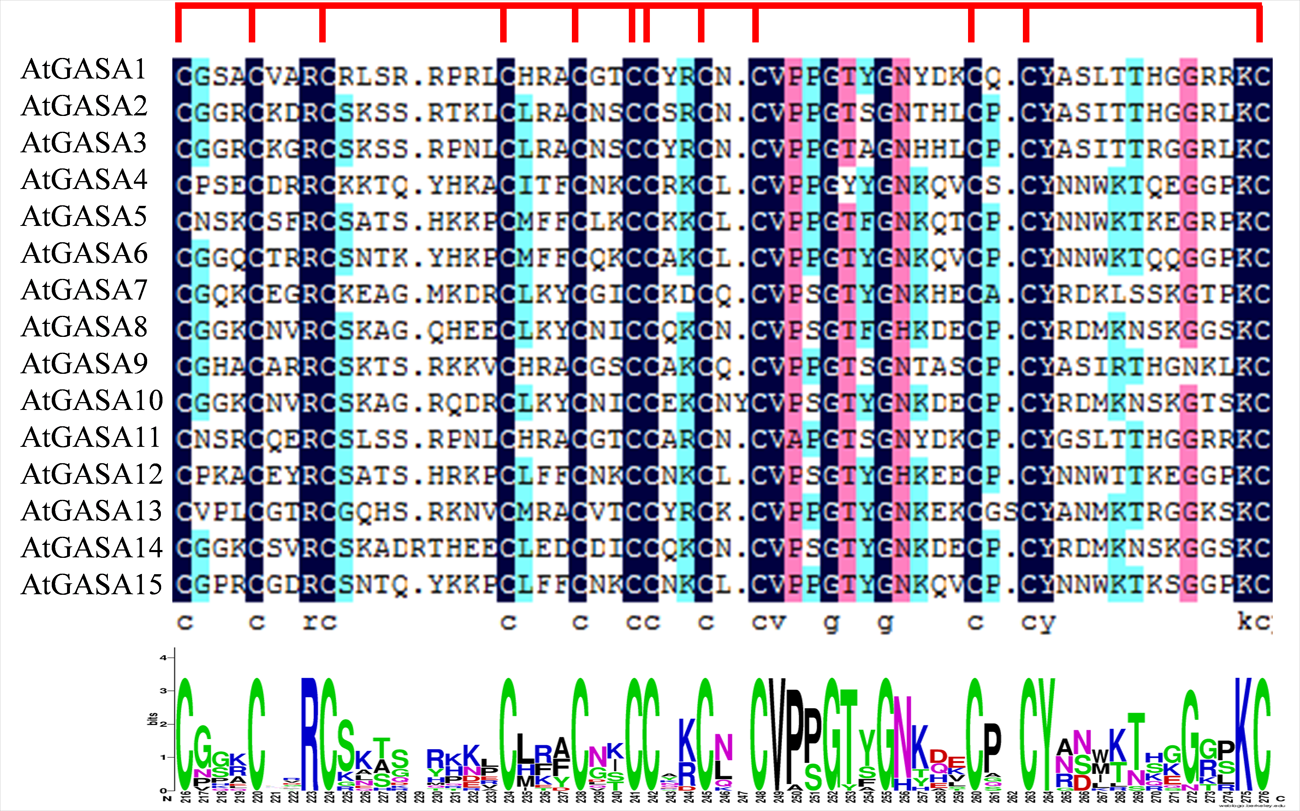

Supplement: Supplementary file 1 — Alignment of GASA domains from AtGASA proteins. (a) Multiple alignments of the AtGASA protein sequences and their conserved GASA domains, red column represented their conserved twelve cysteines. (b) Sequence logo analysis of the conserved AtGASA domains. Each stack represented their amino acids. (TIFF 5642 kb) [file 12864_2017_4213_MOESM1_ESM.tif]

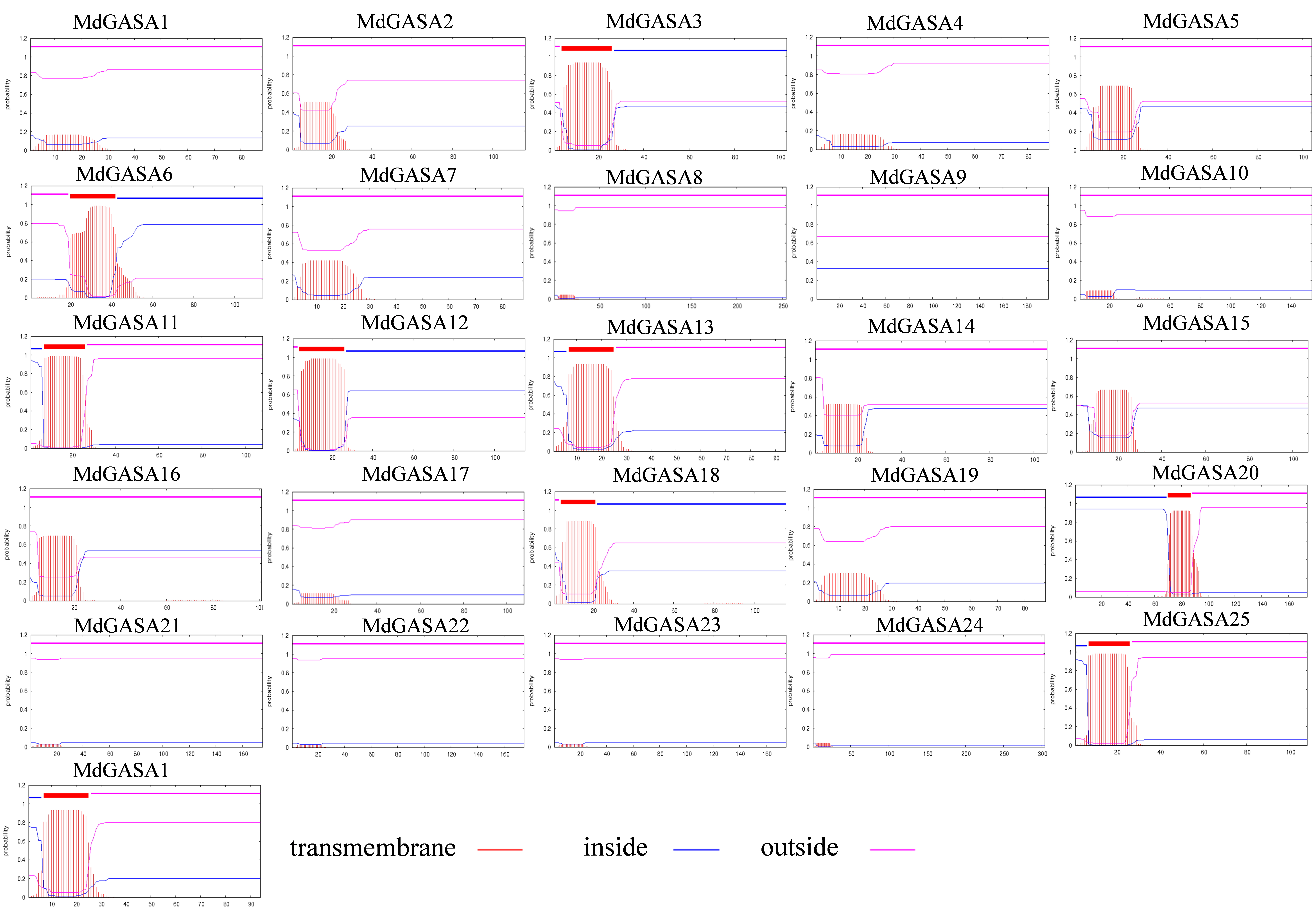

Supplement: Supplementary file 2 — Transmembrane topology analysis of MdGASA proteins. Transmembrane helices of the MdGASA proteins were predicted with the TMHMM server v2.0. The red peaks indicate the predicted transmembrane helices. (TIFF 3853 kb) [file 12864_2017_4213_MOESM2_ESM.tif]

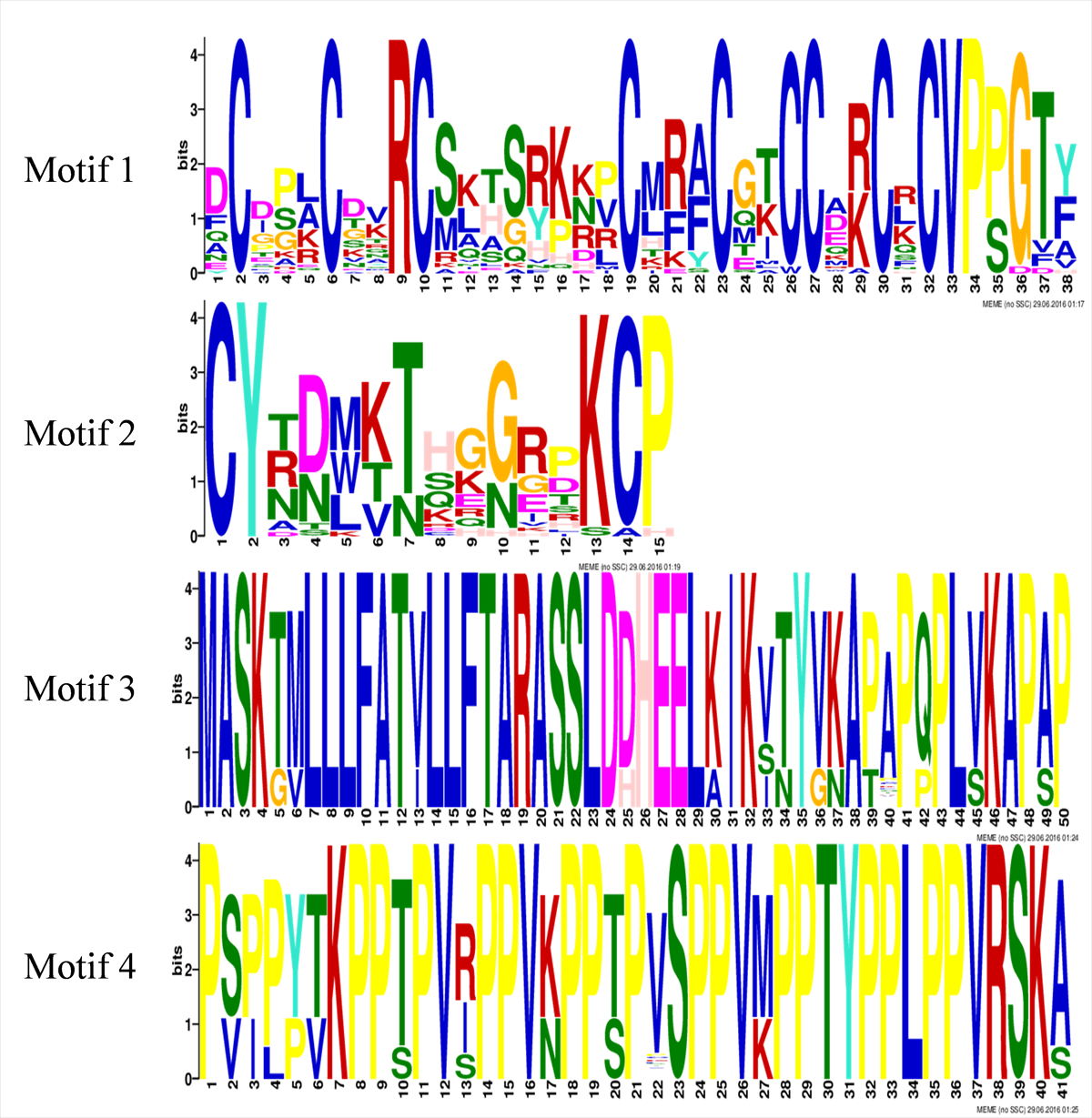

Supplement: Supplementary file 4 — Motif sequence identified by MEME. Motif number was associated with fig. 4c. (TIFF 7247 kb) [file 12864_2017_4213_MOESM4_ESM.tif]

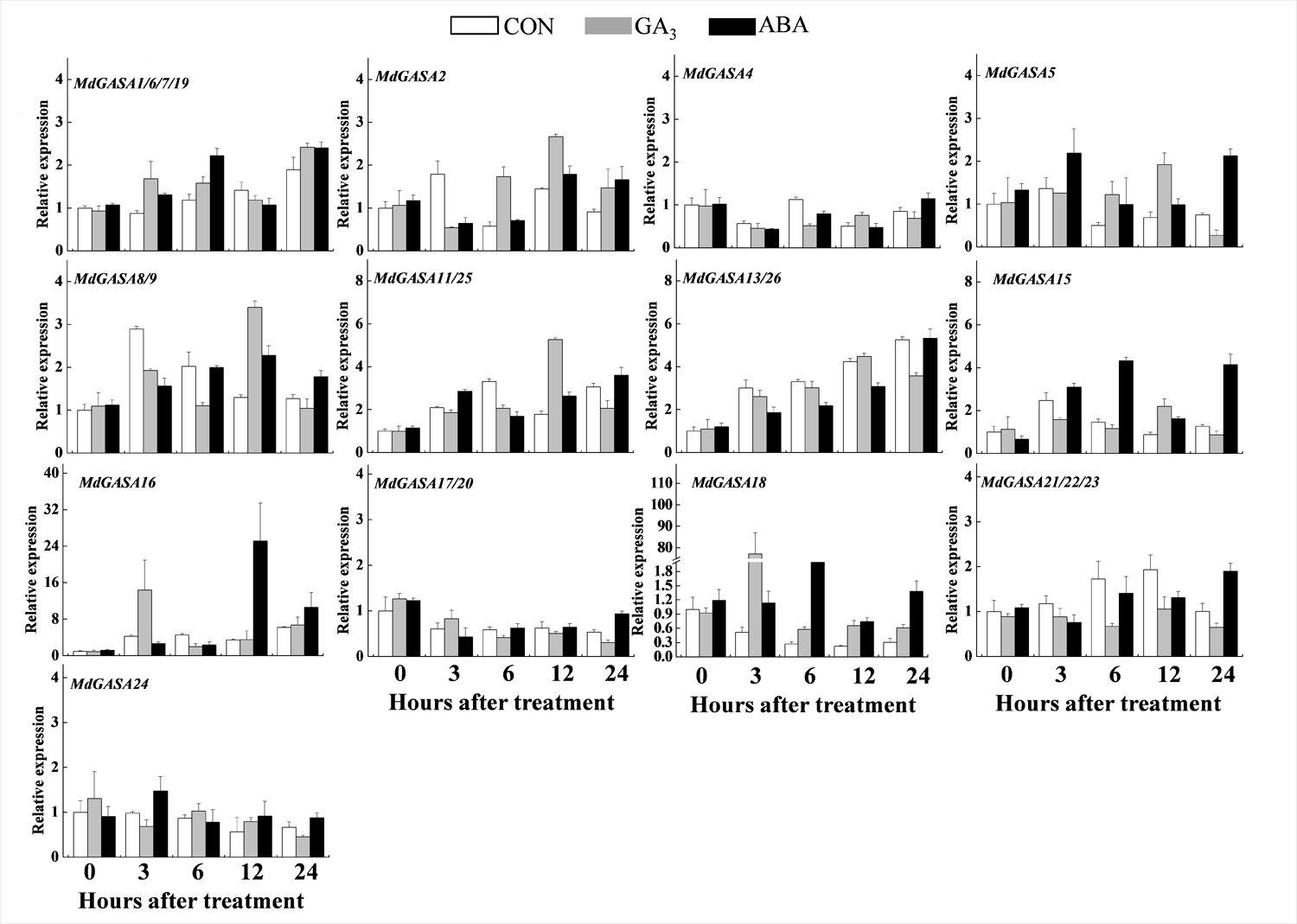

Supplement: Supplementary file 5 — Effects of GA3 and ABA on leaf MdGASA expression levels. Leaves were collected at 0, 1, 3, 6 and 12 h after each treatment. 100Μm GA3, and 300 μM ABA were sprayed in apple leaves. (TIFF 6945 kb) [file 12864_2017_4213_MOESM5_ESM.tif]
