# Supplementary material for: Comprehensive analysis of GASA family members in the Malus domestica genome: identification, characterization, and their expressions in response to apple flower induction
Source: BMC Genomics. 2017 Oct 27;18:827. doi: 10.1186/s12864-017-4213-5 (PMC5658915; doi:10.1186/s12864-017-4213-5)
Supplement: Supplementary file 3 — Secondary structures of MdGASA protein. Their α helix, Extended strand, Random coil and β turn were analyzed. (DOCX 14 kb) [file 12864_2017_4213_MOESM3_ESM.docx]

**Additional file 3** Secondary structures of MdGASA protein.

| Protein | α helix | | Extended strand | | Random coil | | β turn | |
| --- | --- | --- | --- | --- | --- | --- | --- | --- |
|  | Amino acid length | Proportion  (%) | Amino acid length | Proportion  (%) | Amino acid length | Proportion  (%) | Amino acid length | Proportion  (%) |
| MdGASA1 | 22 | 25.00% | 16 | 18.18% | 40 | 45.45% | 10 | 11.36% |
| MdGASA2 | 27 | 23.28% | 15 | 12.93% | 64 | 55.17% | 10 | 8.62% |
| MdGASA3 | 31 | 30.10% | 18 | 17.48% | 48 | 46.40% | 6 | 5.83% |
| MdGASA4 | 21 | 23.86% | 11 | 12.50% | 51 | 57.95% | 5 | 5.68% |
| MdGASA5 | 33 | 31.73% | 11 | 10.58% | 49 | 47.12% | 11 | 10.58% |
| MdGASA6 | 45 | 39.47% | 13 | 11.40% | 50 | 43.86% | 6 | 5.26% |
| MdGASA7 | 25 | 28.41% | 14 | 15.91% | 41 | 46.59% | 8 | 9.09% |
| MdGASA8 | 26 | 10.24% | 28 | 11.02% | 194 | 76.38% | 6 | 2.36% |
| MdGASA9 | 4 | 2.01% | 20 | 10.05% | 169 | 84.92% | 6 | 3.02% |
| MdGASA10 | 29 | 18.83% | 26 | 16.88% | 88 | 57.14% | 11 | 7.14% |
| MdGASA11 | 25 | 23.15% | 11 | 10.19% | 61 | 56.48% | 11 | 10.19% |
| MdGASA12 | 35 | 30.43% | 15 | 13.04% | 55 | 47.83% | 10 | 8.70% |
| MdGASA13 | 32 | 34.04% | 6 | 6.38% | 51 | 54.26% | 5 | 5.32% |
| MdGASA14 | 26 | 24.53% | 15 | 14.15% | 59 | 55.66% | 6 | 5.66% |
| MdGASA15 | 51 | 47.66% | 3 | 2.80% | 44 | 41.12% | 9 | 8.41% |
| MdGASA16 | 25 | 24.75% | 16 | 15.84% | 52 | 51.49% | 8 | 7.92% |
| MdGASA17 | 30 | 27.78% | 19 | 17.59% | 48 | 44.44% | 11 | 10.19% |
| MdGASA18 | 20 | 17.24% | 16 | 13.79% | 76 | 65.52% | 4 | 3.45% |
| MdGASA19 | 29 | 32.95% | 14 | 15.91% | 37 | 42.05% | 8 | 9.09% |
| MdGASA20 | 63 | 36.21% | 25 | 14.37% | 73 | 41.95% | 13 | 7.47% |
| MdGASA21 | 29 | 16.57% | 26 | 14.86% | 112 | 64.00% | 8 | 4.57% |
| MdGASA22 | 29 | 16.57% | 26 | 14.86% | 112 | 64.00% | 8 | 4.57% |
| MdGASA23 | 29 | 16.57% | 26 | 14.86% | 112 | 64.00% | 8 | 4.57% |
| MdGASA24 | 44 | 14.47% | 37 | 12.17% | 212 | 69.74% | 11 | 3.62% |
| MdGASA25 | 24 | 22.22% | 12 | 11.11% | 65 | 60.19% | 7 | 6.48% |
| MdGASA26 | 26 | 27.66% | 6 | 6.38% | 57 | 60.64% | 5 | 5.32% |
