# Supplementary material for: Comprehensive analysis of GASA family members in the Malus domestica genome: identification, characterization, and their expressions in response to apple flower induction
Source: BMC Genomics. 2017 Oct 27;18:827. doi: 10.1186/s12864-017-4213-5 (PMC5658915; doi:10.1186/s12864-017-4213-5)
Supplement: Supplementary file 8 — Sequences of primers used to amplify MdGASA genes and their reference genes. (DOCX 13 kb) [file 12864_2017_4213_MOESM8_ESM.docx]

**Additional file 8** Primer sequences of the *MdGASA* genes and their reference genes.

| Gene | Forward primer (5’-3’) | Reverse primer (5’-3’) | length |
| --- | --- | --- | --- |
| *MdGASA1*/*6*/*7*/*19* | TGAGGCATCAATGGCTGGTTCTCC | AGGGCACTCGTCCTTGTTCCCATA | 160 |
| *MdGASA2* | GCCTCCACCGTAGAAACATC | TAGCCGAGCACCGACTTGTA | 127 |
| *MdGASA3* | TGGCGAGACTCTCATGGTTTCCAA | GGTAGTCACATGCCGCTCCACATT | 126 |
| *MdGASA4* | CGAGGTGTGCAGTAGCAGGAGTTC | TGCCCTTGTCGTTCTTCTTGTCCC | 144 |
| *MdGASA5* | CATGCAGTGCTCGATGTC | CGGGCAGGTCTCAAGATT | 122 |
| *MdGASA8*/9 | CCGCCAACTCCACCACTTACCAAG | AGTGGAGGAGCAGGTGGCTTGTAA | 131 |
| *MdGASA10* | TCCACCACCATCGGCGAATACCA | TTGCAGCGGGCACAGCATAACA | 120 |
| *MdGASA11*/*25* | GCAGCCAGACGCAGTACCACAA | TTGGGTCCTCCTTGCTGGGTCTT | 145 |
| *MdGASA12* | ACTACTGGAGGACACGGATCACCT | TGCCCGATGGAACACAAAGACACT | 148 |
| *MdGASA13*/26 | GCGAGAAGTGCTGTGCCAAATGC | TTGGGTCGTCCTTCCTGGGTCTT | 106 |
| *MdGASA14* | GCAATTCAGATGACCACAACCT | ACAGAAGCATCTCCTACAACAC | 150 |
| *MdGASA15* | GACTTGCTGCCAGCGTTGCA | TCAAGGGCACTTGCGTCTGC | 112 |
| *MdGASA16* | GTGACGGAGCTTGTGTTGTGAGGT | CCAGGTGGAACGCAGAAGCATCTC | 103 |
| *MdGASA17*/20 | CAATCAGAGGTGCAGTAAG | GAGTAGTCATATCAGTGTAGC | 143 |
| *MdGASA18* | AGGCAGCCTTCAACCTCAAGAGTG | GACGCACAAGCACTTGGCACAG | 118 |
| *MdGASA21* | AAGCCACCAACTCCTGTCAGTCC | GCTCTCATGCACACCCTCTTCCTT | 140 |
| *MdGASA24* | AGCCACCTTCAAGATCAAGTCC | ATCCTCTGATTCGATCTCCGTAAG | 122 |
